# Homologous recombination deficiency (HRD) can predict the therapeutic outcomes of immuno-neoadjuvant therapy in NSCLC patients

**DOI:** 10.1186/s13045-022-01283-7

**Published:** 2022-05-18

**Authors:** Zhen Zhou, Zhengping Ding, Jie Yuan, Shengping Shen, Hong Jian, Qiang Tan, Yunhai Yang, Zhiwei Chen, Qingquan Luo, Xinghua Cheng, Yongfeng Yu, Xiaomin Niu, Liqiang Qian, Xiaoke Chen, Linping Gu, Ruijun Liu, Shenglin Ma, Jia Huang, Tianxiang Chen, Ziming Li, Wenxiang Ji, Liwei Song, Lan Shen, Long Jiang, Zicheng Yu, Chao Zhang, Zaixian Tai, Changxi Wang, Rongrong Chen, David P. Carbone, Xuefeng Xia, Shun Lu

**Affiliations:** 1 Department of Medical Oncology, Shanghai Lung Cancer Center, Shanghai Chest Hospital, School of Medicine, Shanghai Jiao Tong University, Shanghai, China; 2 Department of Surgical Oncology, Shanghai Lung Cancer Center, Shanghai Chest Hospital, School of Medicine, Shanghai Jiao Tong University, Shanghai, China; 3 Geneplus-Shenzhen, Shenzhen, China; 4 Department of Oncology, Affiliated Hangzhou First People’s Hospital, Zhejiang University School of Medicine, Hangzhou, China; 5 Geneplus-Beijing, Beijing, China; 6 The Ohio State University Comprehensive Cancer Center, Columbus, OH USA

**Keywords:** Whole-exome sequencing, Homologous recombination deficiency, Neoadjuvant immunotherapy, NSCLC, Biomarker

## Abstract

**Background:**

Neoadjuvant immunotherapy is emerging as novel effective intervention in lung cancer, but study to unearth effective surrogates indicating its therapeutic outcomes is limited. We investigated the genetic changes between non-small cell lung cancer (NSCLC) patients with varied response to neoadjuvant immunotherapy and discovered highly potential biomarkers with indicative capability in predicting outcomes.

**Methods:**

In this study, 3 adenocarcinoma and 11 squamous cell carcinoma NSCLC patients were treated by neoadjuvant immunotherapy with variated regimens followed by surgical resection. Treatment-naive FFPE or fresh tissues and blood samples were subjected to whole-exome sequencing (WES). Genetic alternations were compared between differently-responded patients. Findings were further validated in multiple public cohorts.

**Results:**

DNA damage repair (DDR)-related InDel signatures and DDR-related gene mutations were enriched in better-responded patients, i.e., major pathological response (MPR) group. Besides, MPR patients exhibited provoked genome instability and unique homologous recombination deficiency (HRD) events. By further inspecting alternation status of homology-dependent recombination (HR) pathway genes, the clonal alternations were exclusively enriched in MPR group. Additionally, associations between HR gene alternations, percentage of viable tumor cells and HRD event were identified, which orchestrated tumor mutational burden (TMB), mutational intratumor heterogeneity (ITH), somatic copy number alteration (SCNA) ITH and clonal neoantigen load in patients. Validations in public cohorts further supported the generality of our findings.

**Conclusions:**

We reported for the first time the association between HRD event and enhanced neoadjuvant immunotherapy response in lung cancer. The power of HRD event in patient therapeutic stratification persisted in multifaceted public cohorts. We propose that HR pathway gene status could serve as novel and additional indicators guiding immune-neoadjuvant and immunotherapy treatment decisions for NSCLC patients.

**Supplementary Information:**

The online version contains supplementary material available at 10.1186/s13045-022-01283-7

To the editor

In the past decades, neoadjuvant therapy has provided extra treatment opportunities for lung cancer patients and prolonged their survival [1]. For example, in CheckMate-816 [2] trail assessing efficacy of neoadjuvant nivolumab plus chemotherapy on surgically resectable early-stage NSCLC, the combination led to a MPR rate of 36.9% and pathological complete response (pCR) rate of 24%, significantly outperforms the chemotherapy alone arm. However, very few studies reported effective surrogates indicating the outcomes of neoadjuvant immunotherapy in NSCLC. Herein by performing WES on 14 patients receiving immuno-neoadjuvant therapy, we analyzed samples’ genetic changes and their association with patients’ response, aiming to identify indicators for lung cancer immuno-neoadjuvant therapy.

The clinicopathologic characteristics of the patients enrolled are shown in (Additional file 1: Table S1) with variated ages and stages (Additional file 2: Result S1). All patients received immuno-neoadjuvant therapy but with varied regimen combinations (Additional file 3: Fig. S1A-B). Defined as the time interval between first treatment and surgery, neoadjuvant treatment duration did not exhibit significant difference between different responders (Additional file 3: Fig. S1C). As for mutational analyses, 4243 SNVs and 1290 InDels were identified after filtrations and the mutation signature analysis uncovered the enrichment of DDR-related InDel signatures in MPR group (Additional file 2: Result S2). Besides, 293 mutations in 209 cancer driver genes were detected, with *TP53* (92.9%), *MUC16* (42.9%), *CSMD3* (35.7%) and *MUC4* (35.7%) most frequently mutated (Fig. 1a). No EGFR/ALK/LKB1 mutations were found in patients. Of particular interest, mutations of tumor suppressor genes from DDR and HR pathways were enriched in MPR patients (Fig. 1b, Additional file 2: Result S2), insinuating the occurrence of HRD events in better-responded group.

**Fig. 1:**
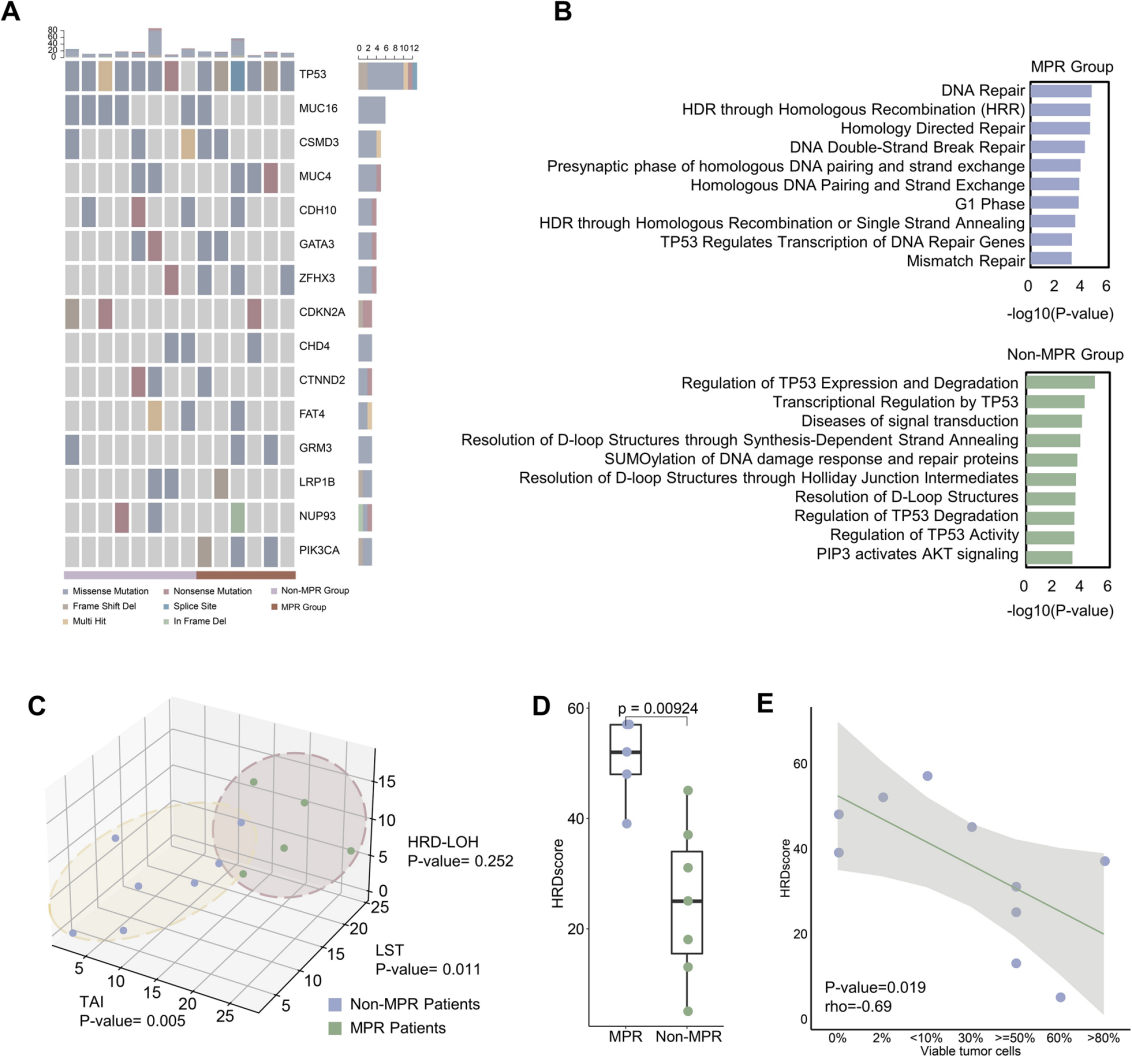
Mutational analysis results between MPR and non-MPR groups. **a** Landscape of frequent SNV and InDel on cancer driver genes. **b** Pathway enrichment analysis on mutated tumor suppressor genes. **c** Three HRD metrics including TAI, LST and HRD-LOH calculated on non-aneuploid samples. **d** HRDscore calculated on non-aneuploid samples. **e** Correlation between HRDscore and percentage of viable tumor cells in all non-aneuploid samples

Consistent with the known provocative effect of altered HR pathway (HR) genes on genome instability, the chromosome and arm-level copy number variation (CNV) burden [3] escalated in MPR group and showed negative correlation with percentage of viable tumor cells after treatment with no histological bias (Additional file 2: Result S3, Additional file 3: Fig. S2A-F). Through SCNA signature quantification in non-aneuploid samples (Additional file 3: Fig. S2K), MPR patients were found enriched for signatures correlated with HRD event, emphasizing the association between HR gene alternations and exacerbated SCNA in MPR subpopulation (Additional file 2: Result S3). Possible association between therapeutic benefits and subclonal SCNAs was also observed (Additional file 2: Result S3). The HRD events in MPR patients were further confirmed by the significantly different quantifications of three HRD-related events, either separately (Fig. 1c) or by the summed HRDscore (Fig. 1d) (Additional file 2: Result S3) and such event enrichment persisted in SCC subtype (Additional file 2: Result S3).

We next inspected TMB and ITH considering their recognized impacts on immunotherapy efficacy [4]. Non-aneuploid and high purity MPR samples carried higher TMB (Fig. 2a), which unsurprisingly demonstrated a negative correlation with residual tumor cells (Fig. 2b). When considering the clonality of mutations, disparity between groups exacerbated (Fig. 2c) and clonal TMB decreased with worse therapeutic response (Fig. 2d). Similar conclusions could be drawn on SCC subtype (Additional file 3: Fig. S4A-D). Intriguingly, apart from lower ITH, clonal somatic mutations in HR pathway were enriched in MPR group regardless of subtypes (Fig. 2e), while such trend was not observed in germline HR gene mutations (Additional file 3: Fig. S4E). We also observed the elevated amount of subclonal SCNA in MPR (Fig. 2F, Additional file 2: Result S4) and the subclonality decreased with residual tumor cells (Fig. 2G, Additional file 2: Result S4). Apart from mutational silence, deletion of 13 core HR genes was concentrated in MPR patients (Fig. 2h) and negatively correlated with residual tumor cells (Fig. 2i), indicating activities of HR genes could be synergistically altered at mutation and copy number level, causing SCNA-level ITH fluctuations, and consequently influence the immuno-neoadjuvant clinical outcomes.

**Fig. 2:**
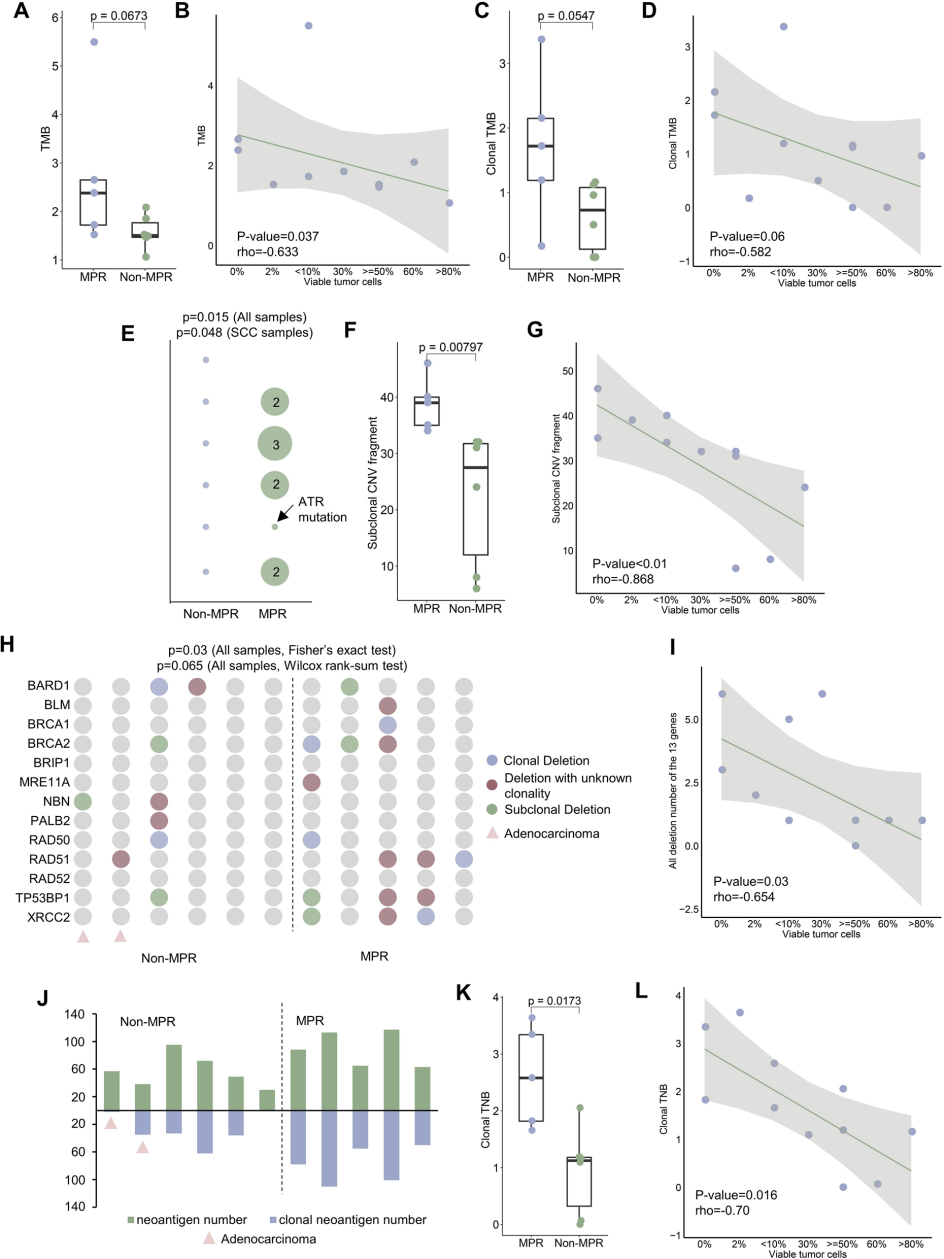
Clonal HR pathway gene deactivation impacts neoadjuvant immunotherapy consequences in NSCLC patients. **a** TMB value distribution between MPR/non-MPR groups. **b** Correlation between TMB and percentage of viable tumor cells. **c** Clonal TMB value in two groups. **d** Correlation between clonal TMB and percentage of viable tumor cells. **e** Existence of HR pathway genes’ clonal SNV in two groups. The size of the circle was proportional to the mutation number. *P* values were calculated by Fisher’s exact test on all and SCC samples. **f** Subclonal SCNA fragment number in two groups. **g** Correlation between subclonal SCNA fragment number and percentage of viable tumor cells. **h** Deletion status of 13 HR core pathway genes in MPR/non-MPR groups. *P* values were calculated by Fisher’s exact test or one-side Wilcoxon rank-sum test. Adenocarcinoma samples were marked with pink triangles. **i** Correlation between focal deletion number of the 13 genes and percentage of viable tumor cells. **j** All and clonal neoantigen number generated by two groups. Adenocarcinoma samples were marked with pink triangles. **k** Clonal TNB value distribution in two groups. **l** Correlation between clonal TNB and percentage of viable tumor cells

Additionally, MPR group generated more neoantigens with higher clonal proportion (Fig. 2j, Additional file 2: Result S5). Clonal TNB measuring neoantigen-level ITH was also significantly higher in MPR and inversely correlated with residual tumor cells (Fig. 2k-l, Additional file 2: Result S5). Since neoantigen load could also be affected by HLA class I genes’ activity, more HLA LOH events were found in non-MPR group and the neoantigens on kept HLA alleles anti-correlated with residual tumor cells (Additional file 2: Result S5). Astoundingly, clonal neoantigens on kept HLA alleles were predominantly resided in the amplified SCNAs for MPR patients (Additional file 2: Result S5), prompting the hypothesis that HR gene mutations could instigate SCNA ITH and HLA LOH and eventually orchestrate to compensate the amount of clonal neoantigen, enhancing the response to immuno-neoadjuvant therapy (Additional file 2: Result S5).

Multi-cohort validation was further conducted to confirm the ability of HR gene alternations in therapeutic outcome prediction. In result, HR pathway mutations occurred more frequently in better-responded immunotherapy patients regardless of the treatment regimen and clinicopathologic characteristics. We also observed higher TMB and longer survival brought by HR mutations in immunotherapy patients as well as the substantial amount of HR pathway alternations in multiracial treatment-free samples, which all countenanced the potential of HRD testing as a novel NSCLC immunotherapy biomarker (Additional file 2: Result S6).

In summary, our study associated the deactivation of HR genes and the resulting HRD event with the improved outcomes of immuno-neoadjuvant treatment in NSCLC and validated our discoveries by public cohorts, offering unprecedented guide prior to the armamentarium of immuno-treatments for NSCLC patients.

## Supplementary Information

**Additional file 1:** Supplementary Tables. **Table S1.** The clinicopathologic characteristics of the 14 patients received immuno-neoadjuvant therapy. **Table S2.** Details of the public cohorts for HRD event validation.

**Additional file 2:** Supplementary Results.

**Additional file 3:** Supplementary Figures. **Figure S1.** Mutation statistics of all curated samples. (A) Regimen combinations of the 14 NSCLC patients. (B) Chemotherapeutic drugs and immunotherapy agents used by each patient. (C) Neoadjuvant immunotherapy duration time distribution between MPR and Non-MPR groups. (D) Correlation between PD-L1 expression and percentage of viable tumor cells in patients. (E) Mutation number retained after filtrations. (F) Mutation number distribution on MPR/Non-MPR and FFPE/Frozen specimen. (G) Six substitution type spectrum plot in two patient groups. P-values were shown above each substitution category. (H) Exposures of three types of mutation signature to known database. SBS: single base substitution; ID: InDel; DBS: double base substitution. Either sum or log2 fold change of the absolute exposures was addedly depicted. (I) Percentage of altered tumor suppressor genes enriched in multiple DNA repair-related pathways in MPR and Non-MPR group. **Figure S2.** SCNA statistical and signature analyses results in groups with distinct therapeutic response. (A) Chromosome level CNV burden between MPR/Non-MPR groups. (B) Arm level CNV burden in two groups. (C) Chromosome level CNV burden between squamous cell carcinoma (SCC) MPR/Non-MPR patients. (D) Arm level CNV burden in two SCC patient groups. (E) Correlation between chromosome level CNV burden and percentage of viable tumor cells. (F) Correlation between arm level CNV burden and percentage of viable tumor cells. (G) Percentage of genome amplified in two groups. (H) Percentage of genome deleted in two groups. (I) Percentage of genome amplified in SCC patients. (J) Percentage of genome deleted in SCC patients. (K) SCNA heatmap only plotting copy number alternations exceeding the threshold +/-0.2. Two aneuploid samples were marked by pink circles. (L) Contributions of three SCNA signatures in all samples. Adenocarcinoma samples were marked with pink triangles. (M) Proportion of SCNA Signature 1 in patients. (N) Proportion of SCNA Signature 7 in patients. (O) Proportion of SCNA Signature 5 in patients. (P) Proportion of SCNA Signature 1 in SCC patients. (Q) Proportion of SCNA Signature 7 in SCC patients. (R) Proportion of SCNA Signature 5 in SCC patients. **Figure S3.** HRDscore in non-aneuploid SCC samples. (A) HRDscore calculated on non-aneuploid SCC samples. (B) Correlation between HRDscore and percentage of viable tumor cells in all non-aneuploid SCC samples. **Figure S4.** Multidimensional ITH comparison results between MPR/Non-MPR groups. (A) TMB value distribution in SCC samples. (B) Correlation between TMB and percentage of viable tumor cells in SCC samples. (C) Clonal TMB value in two groups, only selecting SCC patients. (D) Correlation between clonal TMB and percentage of viable tumor cells on SCC samples. (E) Germline HR pathway gene mutation number in MPR/Non-MPR groups. (F) Subclonal SCNA fragment number in two groups, only selecting SCC samples. (G) Correlation between subclonal SCNA fragment number and percentage of viable tumor cells, only selecting SCC samples. **Figure S5.** Investigation on the neoantigen load difference between patients with distinct outcomes. (A) TNB in two groups. (B) Correlation between TNB values and percentage of viable tumor cells. (C) TNB in two groups, only selecting SCC samples. (D) Correlation between TNB values and percentage of viable tumor cells, only selecting SCC samples. (E) Clonal TNB value distribution in two groups, only selecting SCC samples. (F) Correlation between clonal TNB and percentage of viable tumor cells, only selecting SCC samples. (G) HLA LOH frequency in MPR/Non-MPR groups. (H) TNB value on kept HLA alleles. (I) Correlation between TNB on kept HLA alleles and percentage of viable tumor cells. (J) TNB value on kept HLA alleles, only selecting SCC samples. (K) Correlation between TNB on kept HLA alleles and percentage of viable tumor cells, only selecting SCC samples. (L) Clonal TNB value on kept HLA alleles. (M) Correlation between clonal TNB on kept HLA alleles and percentage of viable tumor cells. (N) Clonal TNB value on kept HLA alleles, only selecting SCC samples. (O) Correlation between clonal TNB on kept HLA alleles and percentage of viable tumor cells, only selecting SCC samples. **Figure S6.** Comprehensive analysis on clonal neoantigen and SCNA bring insights to the function of SCNA on neoantigen generation. (A) Percentage of kept clonal neoantigens on SCNA regions in two groups. (B) Correlation between CNV-related neoantigen proportion and percentage of viable tumor cells. (C) Percentage of kept clonal neoantigens on SCNA regions in two groups, only selecting SCC samples. (D) Correlation between CNV-related neoantigen proportion and percentage of viable tumor cells, only selecting SCC samples. (E) Number of clonal neoantigen on kept HLA alleles and their distribution on 3 types of SCNAs. Adenocarcinoma samples were marked with pink triangles. The sample with ATR mutation was marked with arrow. (F) Correlation between clonal neoantigen number on amplified segments and percentage of viable tumor cells. (G) Correlation between clonal neoantigen number on amplified segments and percentage of viable tumor cells, only selecting SCC samples. (H) Correlation values on all 11 non-aneuploid and high purity samples, calculated by Spearman Correlation Coefficient (SCC). (I) Similar with (H), the correlation values were on MPR samples. (J) Similar with (H), the correlation values were on Non-MPR samples. **Figure S7.** Investigation on HR pathway gene alternation frequency in patients with distinct response from public cohorts. (A) HR pathway gene mutation frequency in J Immunother Cancer. 2020 neoadjuvant dataset. Left: in pCR patients. Middle: in other patients. Right: percentage of distinct mutant reads for each HR gene mutation detected. Mutations from pCR patients were marked with red color. (B) HR pathway gene mutation frequency in N Engl J Med. 2018 neoadjuvant dataset. Left: in MPR patients. Middle: in Non-MPR patients. Right: percentage of distinct mutant reads for each HR gene mutation detected. Mutations from MPR patients were marked with red color. (C) HR pathway gene mutation frequency in DCB patients from Nat Genet. 2018 dataset. Left: all and clonal HR gene mutations. Right: all and clonal HR core pathway mutations. (D) Similar with (C), but using non-DCB patients. (E) Similar with (C), but using J Clin Oncol. 2018 dataset. (F) Similar with (C), but using NDB patients. (G) HR pathway gene mutation frequency in patients achieved DCB in chemotherapy from Cancer Discov. 2017 dataset. (H) Similar with (G), but in NDB patients. (I) HR pathway gene SCNA frequency in patients achieved DCB in chemotherapy from Cancer Discov. 2017 dataset. Left: HR gene SCNAs. Right: HR core pathway gene SCNAs. (J) Similar with (I), but in NDB patients. (K) HR pathway gene mutation frequency in patients achieved CR and PR in chemotherapy from Nat Med. 2018 dataset. Left: all and clonal HR gene mutations. Right: all and clonal HR core pathway mutations. (L) Similar with (K), but in other patients. (M) Similar with (K), but using patients received immunotherapy. (N) Similar with (M), but in other patients. **Figure S8.** Comparisons on TMB values stratified by therapeutic response and HR pathway gene status from public cohorts. (A) TMB and clonal TMB value distribution between DCB and other patients in non-squamous population from Nat Genet. 2018 dataset. Left: TMB. Right: clonal TMB. (B) Similar with (A) but using HR pathway gene clonal mutations as the stratification strategy. (C) TMB value distribution between DCB and NDB patients in J Clin Oncol. 2018. (D) Similar with (C) but focusing on different histological subtypes. Left: in adenocarcinoma subgroup. Right: in squamous subgroup. (E) Similar with (D) but using HR pathway gene mutation as the classification strategy. (F) Clonal TMB value distribution between patients stratified by HRD event in J Clin Oncol. 2018 dataset. Left: between patients with and without HR pathway gene mutation. Right: between patients with and without HR pathway gene clonal mutation. (G) TMB value distribution between CR+PR and other immunotherapy patients in Nat Med. 2018 dataset. (H) Similar with (G) but focusing on different histological subtypes. Left: in non-squamous subgroup. Right: in squamous subgroup. (I) TMB value distribution between CR+PR patients with HRD and other CR+PR patients in Nat Med. 2018. (J) Similar with (F), but in Nat Med. 2018 dataset. **Figure S9.** Survival analyses and TMB comparisons on HRD event-stratified patients in multiple public cohorts. (A) PFS of patients stratified by HR gene mutational condition in Nat Genet. 2018 dataset. Left: between patients with and without HR gene mutation. Right: between patients with and without HR gene clonal mutation. (B) Similar with (A), but on OS data. (C) Similar with (A) but in all patients from J Clin Oncol. 2018 cohort. (D) Similar with (C) but in adenocarcinoma patients. (E) Similar with (C), but in squamous patients. (F) PFS and TMB of all DCB patients with and without HR gene mutations in Nat Genet. 2018 cohort. (G) Similar with (F) but on OS data. (H) Similar with (F) but using HR pathway gene clonal mutations as the classification strategy. (I) Similar with (H) but on OS data. (J) Similar with (F) but in patients from J Clin Oncol. 2018 cohort. **Figure S10.** Survival analyses and TMB comparisons conducted on Nat Med. 2018 blood cohort. (A) Survival and TMB of all chemo-treated non-squamous patients with and without HR gene mutations. Left: OS. Middle: PFS. Right: TMB distribution. (B) Similar with (A) but on squamous patients. (C) Similar with (A) but on patients received immunotherapy. (D) Similar with (C) but on squamous patients. (E) Survival and TMB of all HR pathway-mutant patients treated with immunotherapy and chemotherapy. Left: OS. Middle: PFS. Right: TMB distribution. (F) Similar with (E) but among patients with HR gene clonal mutations. **Figure S11.** Investigation on HR pathway gene alternation frequency and HRD events in multiracial untreated patients. (A) HR pathway gene mutation frequency in Sci Rep. 2015 dataset. Left: all and clonal HR gene mutations. Right: all and clonal HR core pathway mutations. (B) Similar with (A), but in J Thorac Oncol. 2020 cohort. (C) Similar with (A), but in TCGA-LUAD dataset. (D) Similar with (A), but in TCGA-LUSC dataset. (E) HR pathway gene alternation frequency in TCGA-LUAD dataset. (F) Similar with (E), but in TCGA-LUSC dataset. (G) Correlation between CNV burden and HRDscore in TCGA-LUAD dataset. (H) Similar with (G), but in TCGA-LUSC dataset.

**Additional file 4:** Supplementary Materials and methods.

## Data Availability

Data generated in the current study are available from the corresponding author on reasonable request. Data used in the validation analyses were either downloaded from the supplemental files of the publications or from cBioPortal database.

## Abbreviations

CNV: Copy number variation
DDR: DNA damage repair
HR: Homology-dependent recombination
HRD: Homologous recombination deficiency
InDel: Small insertions and deletion
ITH: Intratumor heterogeneity
MPR: Major pathological response
NSCLC: Non-small cell lung cancer
SCC: Squamous cell carcinoma
SCNA: Somatic copy number alteration
SNV: Somatic single nucleotide variant
TMB: Tumor mutational burden
TNB: Tumor neoantigen burden
WES: Whole-exome sequencing
