# Supplementary material for: Homologous recombination deficiency (HRD) can predict the therapeutic outcomes of immuno-neoadjuvant therapy in NSCLC patients
Source: J Hematol Oncol. 2022 May 18;15:62. doi: 10.1186/s13045-022-01283-7 (PMC9118717; doi:10.1186/s13045-022-01283-7)
Supplement: Supplementary file 2 — Additional file 2: Supplementary Results. [file 13045_2022_1283_MOESM2_ESM.docx]

**Supplementary Results**

**Result S1: Minor difference was observed in clinicopathological characteristics between patients with distinct response to therapy**

Patients enrolled were categorized as MPR using definition described in Supplementary Materials and methods. The median age of MPR/Non-MPR group was 62 (range, 55-68) and 60 (range, 44-68) accordingly with no sex differences. Most of the MPR patients were diagnosed with stage IIIA NSCLC while stage IIIA/IIIB patients were evenly distributed in Non-MPR group. The median treatment duration time was 83 (69-97) and 71 (50-92) days respectively. No correlation was observed between PD-L1 expression and percent of viable tumor cells (Suppl. Fig. 1D).

**Result S2: Mutational analysis identified the preponderance of homologous recombination-related genes in MPR group**

A total of 11598 SNVs and InDels were identified by WES, while 4243 and 1290 mutations retained through filtrations (Suppl. Fig. 1E). No obvious number difference was observed between MPR/Non-MPR and FFPE/Frozen tissues (Suppl. Fig. 1F). Mutation signature analysis was also conducted on unfiltered mutations to decipher the possible etiological reasons behind distinct therapeutic response. As shown in Suppl. Fig. 1G, the substitution spectrum plot displayed insignificant statistically difference between groups. By further mapping mutations to known mutational signatures in COSMIC database (accessed in March 2021), SBS 4 associated with tobacco-smoking-induced cancer contributed to high proportions of mutations in MPR/non-MPR groups (Suppl. Fig. 1H), consistent with their smoking history. Sorted by log2 contribution fold change between MPR/Non-MPR groups, InDel signatures ID6 and ID8 preponderated in MPR patients, both were attributed to DNA damage repair (DDR) mechanisms (Suppl. Fig. 1H). Regarding the relatively low absolute exposure value to known features, no obvious enrichment was observed on DBS signatures. As for the pathway enrichment of mutated tumor suppressor genes, Reactome pathways including R-HSA-5693567 and R-HSA-6796648 demonstrated significant difference between MPR and Non-MPR groups (Suppl. Fig. 1I).

**Result S3: MPR group exhibited exacerbated somatic copy number alternations and occurrence of HRD events**

Escalation of the chromosome and arm level CNV burden was observed for all MPR (Suppl. Fig. 2A-B) and squamous cell carcinoma (SCC) MPR patients (Suppl. Fig. 2C-D). The two CNV burdens also showed negative correlation with percent of viable tumor cells (Suppl. Fig. 2E-F). Percent of amplified or deleted genome also manifested an increased trend in MPR subpopulation for all 14 patients (Suppl. Fig. 2G-H) and SCC subtype (Suppl. Fig. 2I-J).

As for the SCNA signature analysis, the signature enrichments were computed by CNsignatures tool and compared between MPR and Non-MPR patients. Three signatures exhibited different enrichment in the two groups (Suppl. Fig. 2L). According to previous results from ovarian carcinoma^1^, the SCNA Signature 1 was anti-correlated with HRD SNV signature while SCNA Signature 7 showed positive association with HRD SNV signature 3. Unsurprisingly, the group-wise SCNA signature distributions showed high consistency with the mutation status of HR-related genes in MPR/Non-MPR patients (Suppl. Fig. 2M-N), i.e. lower Signature 1 and higher Signature 7 proportion in MPR group. Additionally, SCNA Signature 5, which was associated with subclonal copy number changes, got significantly elevated proportion in MPR group, raising the presumption that HR gene deactivation escalated SCNA level intratumor heterogeneity (ITH) in MPR group. When narrowing to SCC patients, the SCNA signature disparities reduced (Suppl. Fig. 2P-R). Considering copy number variation could be originated from multiple mechanisms including non-allelic homologous recombination (NAHR), non-homologous end joining (NHEJ), DNA replication error and meiotic processes^2^, these analyses on SCNA signatures accentuated the association between HR pathway gene alternations and exacerbated SCNA in the patients achieved MPR.

As for the HRD event quantification, three HRD-related events including telomeric allelic imbalance (TAI), large-scale state transitions (LST) and loss of heterozygosity (LOH) in non-aneuploid samples were compared between MPR/Non-MPR groups. Additionally, the summed HRDscore in MPR group was significantly higher and negatively correlated with percent of viable tumor cells after treatment, both on all non-aneuploid samples (Fig. 1D-E) and SCC samples (Suppl. Fig. 3A-B).

**Result S4:** **Intratumor heterogeneity divergence caused by HRD potentially engendered the therapeutic response difference between two groups**

Previous analysis on SCNA signatures implicated the possible existence of subclonal SCNA in MPR group, we further confirmed the conjecture by selecting the SCNA from non-aneuploid and high purity samples and annotating them with clonality. With increased percentage of viable tumor cells in the sample, the subclonal SCNA fragment number manifested a decreased trend in all and SCC subpopulation (Suppl. Fig. 4F-G). Given that HRD essentially leads to the occurrence of recurrent SCNAs^3^, the clonal HR pathway mutations preponderated in MPR group could possibly be the source of these SCNA ITH.

**Result S5: Investigation on the neoantigen generation in MPR and Non-MPR groups**

Analyses on neoantigens were conducted after discarding aneuploid or low purity samples. Apart from the total neoantigen amount, TNB values in MPR group was unsurprisingly higher for all patients (Suppl. Fig. 5A) and negatively correlated with percentage of viable tumor cells (Suppl. Fig. 5B). These trends persisted in SCC subpopulation (Suppl. Fig. 5C-D). Moreover, the clonal TNB for SCC samples demonstrated a higher trend in MPR group (Suppl. Fig. 5E) and inversely correlated with percent of viable tumor cells (Suppl. Fig. 5F), which could possibly caused by low mutational level ITH.

When incorporating the HLA LOH status in neoantigen analysis, we found higher LOH frequency of HLA-A, HLA-B and HLA-C loci in Non-MPR group (Suppl. Fig. 5G). By further retaining neoantigens on kept HLA alleles, MPR patients exhibited higher neoantigen load (Suppl. Fig. 5H). Again, the amount of the kept neoantigens decreased with percentage of viable tumor cells (Suppl. Fig. 5I) and such tendency persevered in SCC patients (Suppl. Fig. 5J-K). As for low-ITH neoantigens on kept HLA alleles, differences between MPR and Non-MPR group exacerbated in all patients (Suppl. Fig. 5L) or SCC population (Suppl. Fig. 5N). Analogous correlation trend between kept clonal TNB and quantified therapeutic response was also observed (Suppl. Fig. 5M, O). Astoundingly, the HLA-retained clonal neoantigens were mostly resided in the regions with somatic copy number alternations in MPR group (Suppl. Fig. 6A) and these CNV-related neoantigen was associated with percent of variable tumor cells (Suppl. Fig. 6B). The 9 SCC patients also demonstrated a similar trend (Suppl. Fig. 6C-D). When taking the alternation type into account, the clonal neoantigens on kept HLA alleles were predominantly resided in the amplified segments (amplification type SCNA) for MPR patients (Suppl. Fig. 6E), denoting an inner association between clonal TNB and copy number amplification on our samples. The kept clonal neoantigen number on amplified segments demonstrated akin negative correlations with percentage of viable cells regardless of histological subtype (Suppl. Fig. 6F-G). Notably, the only MPR sample without HR gene mutations possessed the highest number of clonal neoantigen (n=110) but they scarcely resided in SCNA segments (Suppl. Fig. 6E), raising the hypothesis that mutations in HR genes could lead to the increased SCNA-level ITH and LOH of HLA, and eventually orchestrate to compensate the amount of clonal antigen burden in patients. Indeed, strong association between HRD event or HR pathway gene alternation and key determinants of neoadjuvant immunotherapy outcome existed in all 11 non-aneuploid and high purity samples (Suppl. Fig. 6H). Moreover, HRD event correlated well with clonal neoantigen generation and CNV-related clonal neoantigens in MPR samples (Suppl. Fig. 6I) and such correlations alleviated in Non-MPR patients (Suppl. Fig. 6J).

**Result S6: Multi-cohort validation confirmed the feasibility of HRD mutational testing in immunotherapy patient stratification**

Multiple public NSCLC therapeutic cohorts were selected to validate the power of HR pathway gene in patient stratification. More detailedly, data from two neoadjuvant immunotherapy cohorts, two immunotherapy cohorts and two cohorts containing patients received distinct therapy types were curated (details in Additional File 4: Supplementary Materials and methods). As shown in Supplementary Table 2 (Additional File 1), the selected datasets variated in therapeutic regimens and treatment design. To begin with, we firstly scrutinized the alternation frequency of HR pathway genes in distinctly-responded patients from different public cohorts. For J Immunother Cancer. 2020 dataset from the two neoadjuvant studies, all patients (100%) achieved pCR had HR pathway gene mutation (Suppl. Fig. 7A, left) while the HR gene mutation was sporadic in other patients (Suppl. Fig. 7A, middle, 28.5% patients). When inspecting HR gene mutation clonality, pCR patients possessed mutations with higher percentage of distinct mutant reads (Suppl. Fig. 7A, right), indicating the lower HR gene mutational ITH in better-responded subgroup. Similar high prevalence of HR gene mutations in MPR patients (Suppl. Fig. 7B, left, 66.7% patients), the HR gene mutational disperse in Non-MPR group (Suppl. Fig. 7B, middle, 25% patients) as well as lower HR gene-related ITH (Suppl. Fig. 7B, right) were observed in N Engl J Med. 2018 neoadjuvant dataset. As for the immunotherapy datasets, after addedly considering mutational ITH by annotating mutations with somatic clonal probability>=0.8 as clonal, DCB patients from Nat Genet. 2018 cohort significantly carried more clonal HR gene mutations (P-value=0.045, Fisher’s exact test) and clonal HR core pathway gene mutations (P-value=0.08, Fisher’s exact test) than other patients (Suppl. Fig. 7C-D). Interestingly, such discrepancy was not observed in targeted sequencing immunotherapy dataset J Clin Oncol. 2018 (Suppl. Fig. 7E-F), denoting the prominence of panel design in targeted sequencing when using HR pathway genes’ mutational status as immunotherapy biomarkers. In addition, for patients from Cancer Discov. 2017, no obvious proportional difference on mutated HR genes was observed between patients with distinct response to chemotherapy (Suppl. Fig. 7G-H), denoting the vague connection between HR gene mutation and chemotherapy response in NSCLC. The focal SCNA frequency of HR pathway genes among these patients exhibited an analogous trend (Suppl. Fig. 7I-J). Lastly, for the blood-based targeted sequencing dataset Nat Med. 2018, patients achieved complete response (CR) or partial response (PR) in chemotherapy and other chemo-treated patients showed similar HR gene mutation frequency (Suppl. Fig. 7K-L). When inspecting patients received immunotherapy, HR gene mutational prevalence increased slightly in CR+PR (CR and PR) patients (Suppl. Fig. 7M-N). In conclusion, multi-cohort validation confirmed the increased HR pathway gene mutational frequency in patients better-responded to immunotherapy while HR genes demonstrated negligible connection with therapeutic outcomes in chemo-treated NSCLC patients. When applying HR pathway gene alternations as immunotherapy biomarkers in targeted sequencing, extra attention should be given to panel design.

Additionally, regarding the availability of data, comparisons on genomic characteristics were further conducted between patients stratified by therapeutic response and HR pathway gene conditions. More specifically, two immunotherapy datasets including Nat Genet. 2018, J Clin Oncol. 2018 and Nat Med. 2018 cohort containing chemo/immuno-treated subgroups were selected for comparisons on TMB and patient survival. To begin with, among the non-squamous patients from Nat Genet. 2018 dataset, we observed elevated TMB and clonal TMB values in those with better curative effect (Suppl. Fig. 8A) or HR gene clonal mutations (Suppl. Fig. 8B), endorsing the power of HRD testing in therapeutic effect prediction. As for DCB and NDB patients from J Clin Oncol. 2018 cohort, though TMB elevated in DCB subpopulation (Suppl. Fig. 8C), it failed to distinguish SCC patients with different response (Suppl. Fig. 8D, right). When inspecting patients with and without HR gene mutations, the TMB disparity increased for both adenocarcinoma (Suppl. Fig. 8E, left) and squamous (Suppl. Fig. 8E, right) patients. Moreover, patients with HR gene mutations in J Clin Oncol. 2018 cohort possessed higher clonal TMB (Suppl. Fig. 8F, left) while HR gene clonal mutations (defined by Taf>=0.3) prioritized patients with significantly higher clonal TMB (Suppl. Fig. 8F, right). Finally, for immunotherapy patients in the biomarker-evaluable population of Nat Med. 2018 dataset, CR+PR patients got significantly higher TMB value (Suppl. Fig. 8G) but TMB again failed to stratify the better-responded patients in SCC subpopulation (Suppl. Fig. 8H, right). Intriguingly, CR+PR patients with HRD bore higher TMB than other CR+PR patients (Suppl. Fig. 8I) and patients with HR gene global or clonal (defined by Taf>=0.05) mutations in blood unsurprisingly harbored higher clonal TMB (Suppl. Fig. 8J). The above comprehensive analysis on TMB confirmed their substantial elevation in HRD patients receiving diversified non-neoadjuvant treatment regimens and possessing varying clinicopathologic characteristics. Regarding higher TMB correlates with better-responded NSCLC patients, our observations sustained the feasibility of using HRD or HR gene mutations as novel immunotherapy biomarker in NSCLC.

HR gene mutational information was further incorporated in classification to study the survival disparateness on public cohorts. For the patients from Nat Genet. 2018 cohort, subgroups with HR gene global and clonal mutations exhibited better progression-free survival (PFS) (Suppl. Fig. 9A). Similar observations could be made on overall survival (OS) (Suppl. Fig. 9B) data. As for the targeted-sequencing cohort J Clin Oncol. 2018, patients possessing HR gene mutations consonantly got better PFS (Suppl. Fig. 9C) but incongruence on survival existed between adenocarcinoma (Suppl. Fig. 9D) and squamous (Suppl. Fig. 9E) patients. Again, when applying HR gene mutation as immunological biomarker in targeted sequencing, the sequencing panel should be elaborately designed. Additionally, DCB patients possessing HR pathway gene mutations surprisingly evinced better PFS and higher TMB than other DCB patients in Nat Genet. 2018 dataset (Suppl. Fig. 9F) and such trend was analogously observed in OS data (Suppl. Fig. 9G). When narrowing to clonal HR gene mutations, the trend preserved in PFS (Suppl. Fig. 9H) and OS (Suppl. Fig. 9I). Similarly, DCB patients with HRD manifested significantly better survival and higher TMB values than other DCB participants in J Clin Oncol. 2018 (Suppl. Fig. 9J) dataset. Our multi-cohort analyses confirmed that HRD patients achieved generally better survival and the potency of HRD event in survival stratification, even among better responders in immunotherapy, i.e. DCB NSCLC patients.

With advantages including non-invasive, relatively costless, riskless and biopsy bias-reducing, liquid biopsies have received growing attention in tumor early diagnosis, recurrence monitoring and therapeutic guidance. We next conducted survival analysis in the publicly available blood-based Nat Med. 2018 dataset measuring circulating free DNA (cfDNA) to shed light on applying HRD mutational testing in blood biopsy. Firstly, no survival but significant TMB difference was observed between HR mutant and HR wild type chemo-treated patients in non-squamous (Suppl. Fig. 10A) and squamous (Suppl. Fig. 10B) patients, which denoted the uselessness of HRD as therapeutic biomarker in NSCLC chemotherapy treatment. When focusing on patients treated by immunotherapy, HR gene mutant group significantly got higher TMB but inconspicuous group-wise survival difference existed in non-squamous (Suppl. Fig. 10C) and squamous (Suppl. Fig. 10D) subpopulation. Regarding these HRD events were measured by targeted sequencing, different results could possibly be observed by more comprehensive HRD testing strategy. Ultimately, for patients with HR gene global and clonal mutations, patients received immunotherapy exhibited significantly better survival (Suppl. Fig. 10E-F). Apart from prioritizing patients with better immunological response, HRD event could provide suggestions over different therapy types for NSCLC patients.

Last of all, we investigated the mutational frequency of HR genes in multiracial datasets to guarantee the universality of HRD event in untreated NSCLC. The HR and core HR pathway gene global and clonal (defined by Taf>=0.3) mutation frequency in Sci Rep. 2015 Chinese SCC (Suppl. Fig. 11A), J Thorac Oncol. 2020 African American NSCLC (Suppl. Fig. 11B), TCGA-LUAD (Suppl. Fig. 11C) and TCGA-LUSC (Suppl. Fig. 11D) cohorts were calculated. In addition, HR pathway gene alternation information including mutations, deep deletions and epigenetic silencing from a previous paper^4^ were retrieved for TCGA-LUAD (Suppl. Fig. 11E) and TCGA-LUSC (Suppl. Fig. 11F) datasets. Both histological subtypes exhibited around 50% HR gene alternation frequency. The quantified HRD event (HRDscore) additionally demonstrated congruent strong positive correlation with CNV burden in two TCGA lung cancer datasets (Suppl. Fig. 11G-H), again validated the concomitant relationship between HRD event and drastic CNV. Together with discoveries listed above, HRD events occurred more frequently in better-responded patients receiving immunotherapy regardless of the treatment regimen and the clinicopathologic characteristics. We observed the resultant higher TMB and consequently longer survival brought by HRD as well as the substantial amount of HRD events in multiracial samples, which all countenanced the potential of HRD testing as a novel immunotherapy biomarker.

**References**

1. Macintyre, G. *et al.* Copy-number signatures and mutational processes in ovarian carcinoma. *Nat Genet* **50**, 1262–1270 (2018).

2. de Smith, A. J., Walters, R. G., Froguel, P. & Blakemore, A. I. Human genes involved in copy number variation: mechanisms of origin, functional effects and implications for disease. *Cytogenet Genome Res* **123**, 17–26 (2009).

3. Hastings, P., Lupski, J. R., Rosenberg, S. M. & Ira, G. Mechanisms of change in gene copy number. *Nat Rev Genet* **10**, 551–564 (2009).

4. Knijnenburg, T. A. *et al.* Genomic and Molecular Landscape of DNA Damage Repair Deficiency across The Cancer Genome Atlas. *Cell Rep* **23**, 239-254.e6 (2018).
