# Supplementary material for: Homologous recombination deficiency (HRD) can predict the therapeutic outcomes of immuno-neoadjuvant therapy in NSCLC patients
Source: J Hematol Oncol. 2022 May 18;15:62. doi: 10.1186/s13045-022-01283-7 (PMC9118717; doi:10.1186/s13045-022-01283-7)
Supplement: Supplementary file 3 — Additional file 3: Supplementary Figures. Figure S1. Mutation statistics of all curated samples. (A) Regimen combinations of the 14 NSCLC patients. (B) Chemotherapeutic drugs and immunotherapy agents used by each patient. (C) Neoadjuvant immunotherapy duration time distribution between MPR and Non-MPR groups. (D) Correlation between PD-L1 expression and percentage of viable tumor cells in patients. (E) Mutation number retained after filtrations. (F) Mutation number distribution on MPR/Non-MPR and FFPE/Frozen specimen. (G) Six substitution type spectrum plot in two patient groups. P-values were shown above each substitution category. (H) Exposures of three types of mutation signature to known database. SBS: single base substitution; ID: InDel; DBS: double base substitution. Either sum or log2 fold change of the absolute exposures was addedly depicted. (I) Percentage of altered tumor suppressor genes enriched in multiple DNA repair-related pathways in MPR and Non-MPR group. Figure S2. SCNA statistical and signature analyses results in groups with distinct therapeutic response. (A) Chromosome level CNV burden between MPR/Non-MPR groups. (B) Arm level CNV burden in two groups. (C) Chromosome level CNV burden between squamous cell carcinoma (SCC) MPR/Non-MPR patients. (D) Arm level CNV burden in two SCC patient groups. (E) Correlation between chromosome level CNV burden and percentage of viable tumor cells. (F) Correlation between arm level CNV burden and percentage of viable tumor cells. (G) Percentage of genome amplified in two groups. (H) Percentage of genome deleted in two groups. (I) Percentage of genome amplified in SCC patients. (J) Percentage of genome deleted in SCC patients. (K) SCNA heatmap only plotting copy number alternations exceeding the threshold +/-0.2. Two aneuploid samples were marked by pink circles. (L) Contributions of three SCNA signatures in all samples. Adenocarcinoma samples were marked with pink triangles. (M) Proportion of SCNA Signature 1 in [file 13045_2022_1283_MOESM3_ESM.pdf]

Suppl. Fig. 1

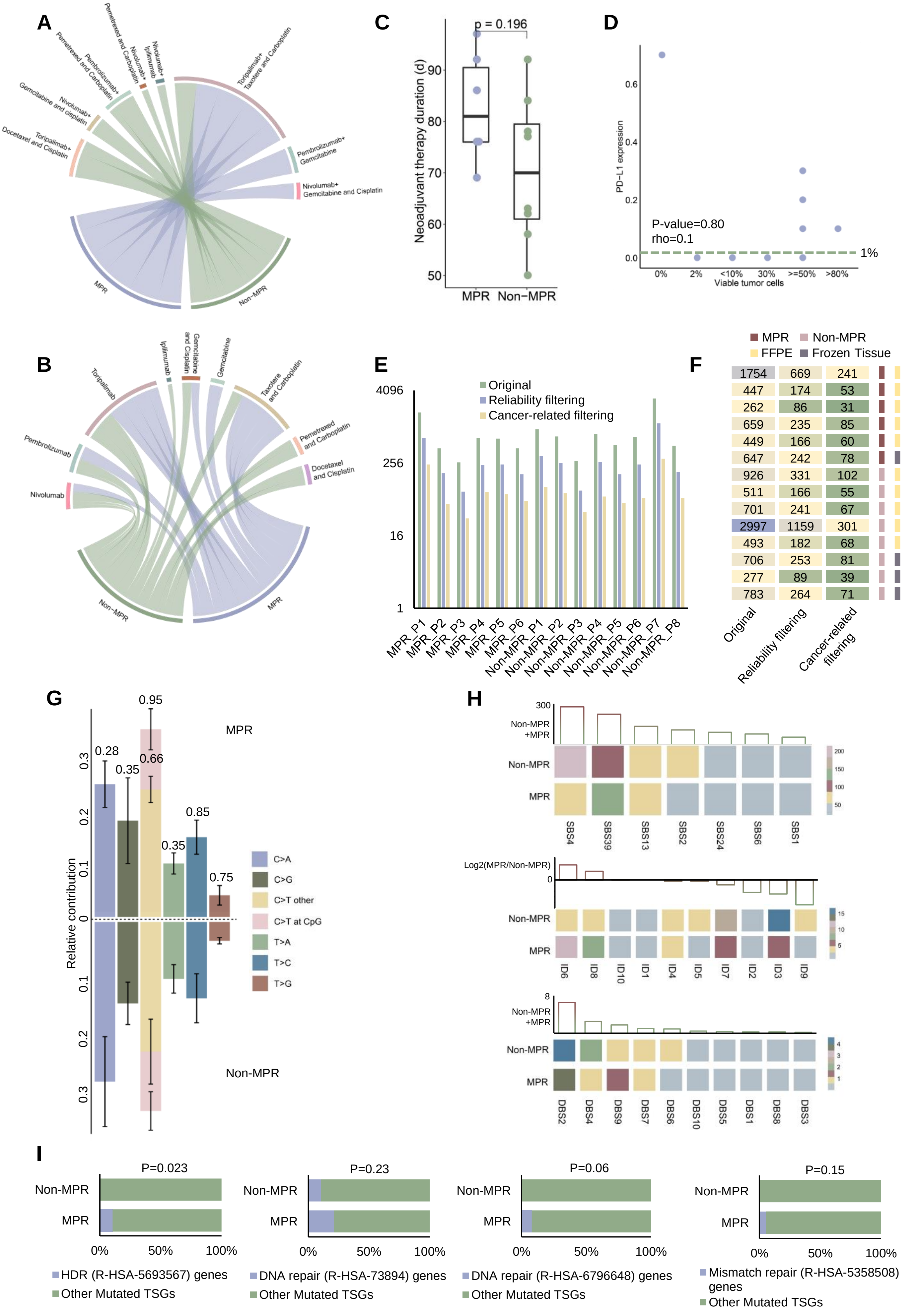

A
Pemetrexed and Carboplatin
Nivolumab+ Ipilimumab
Nivolumab+
Pembrolizumab+ Pemetrexed and Carboplatin
Nivolumab+ Gemcitabine and cisplatin
Toripalimab+ Docetaxel and Cisplatin
Toripalimab+ Taxotere and Carboplatin
Pembrolizumab+ Gemcitabine
Nivolumab+ Gemcitabine and Cisplatin
MPR
Non-MPR
B
Toripalimab
Ipilimumab
Gemcitabine and Cisplatin
Gemcitabine
Taxotere and Carboplatin
Pembrolizumab
Pemetrexed and Carboplatin
Docetaxel and Cisplatin
Nivolumab
Non-MPR
MPR
C
p = 0.196
Neoadjuvant therapy duration (d)
90
80
70
60
50
MPR
Non−MPR
D
PD−L1 expression
0.6
0.4
0.2
0.0
P-value=0.80
rho=0.1
1%
0%
2%
<10%
30%
>=50%
>80%
Viable tumor cells
E
Original
Reliability filtering
Cancer-related filtering
4096
256
16
1
MPR_P1
MPR_P2
MPR_P3
MPR_P4
MPR_P5
MPR_P6
Non-MPR_P1
Non-MPR_P2
Non-MPR_P3
Non-MPR_P4
Non-MPR_P5
Non-MPR_P6
Non-MPR_P7
Non-MPR_P8
F
MPR
Non-MPR
FFPE
Frozen Tissue
1754 669 241
447 174 53
262 86 31
659 235 85
449 166 60
647 242 78
926 331 102
511 166 55
701 241 67
2997 1159 301
493 182 68
706 253 81
277 89 39
783 264 71
Original
Reliability filtering
Cancer-related filtering
G
Relative contribution
0.3
0.2
0.1
0
0.28
0.35
0.95
0.66
0.35
0.85
0.75
MPR
Non-MPR
C>A
C>G
C>T other
C>T at CpG
T>A
T>C
T>G
H
300
Non-MPR +MPR
Non-MPR
MPR
SBS4
SBS39
SBS13
SBS2
SBS24
SBS6
SBS1
200
150
100
50
Log2(MPR/Non-MPR)
0
ID6
ID8
ID10
ID1
ID4
ID5
ID7
ID2
ID3
ID9
15
10
5
8
DBS2
DBS4
DBS9
DBS7
DBS6
DBS10
DBS5
DBS1
DBS8
DBS3
4
3
2
1
I
P=0.023
P=0.23
P=0.06
P=0.15
Non-MPR
MPR
0%
50%
100%
HDR (R-HSA-5693567) genes
Other Mutated TSGs
DNA repair (R-HSA-73894) genes
DNA repair (R-HSA-6796648) genes
Mismatch repair (R-HSA-5358508) genes

Suppl. Fig. 2

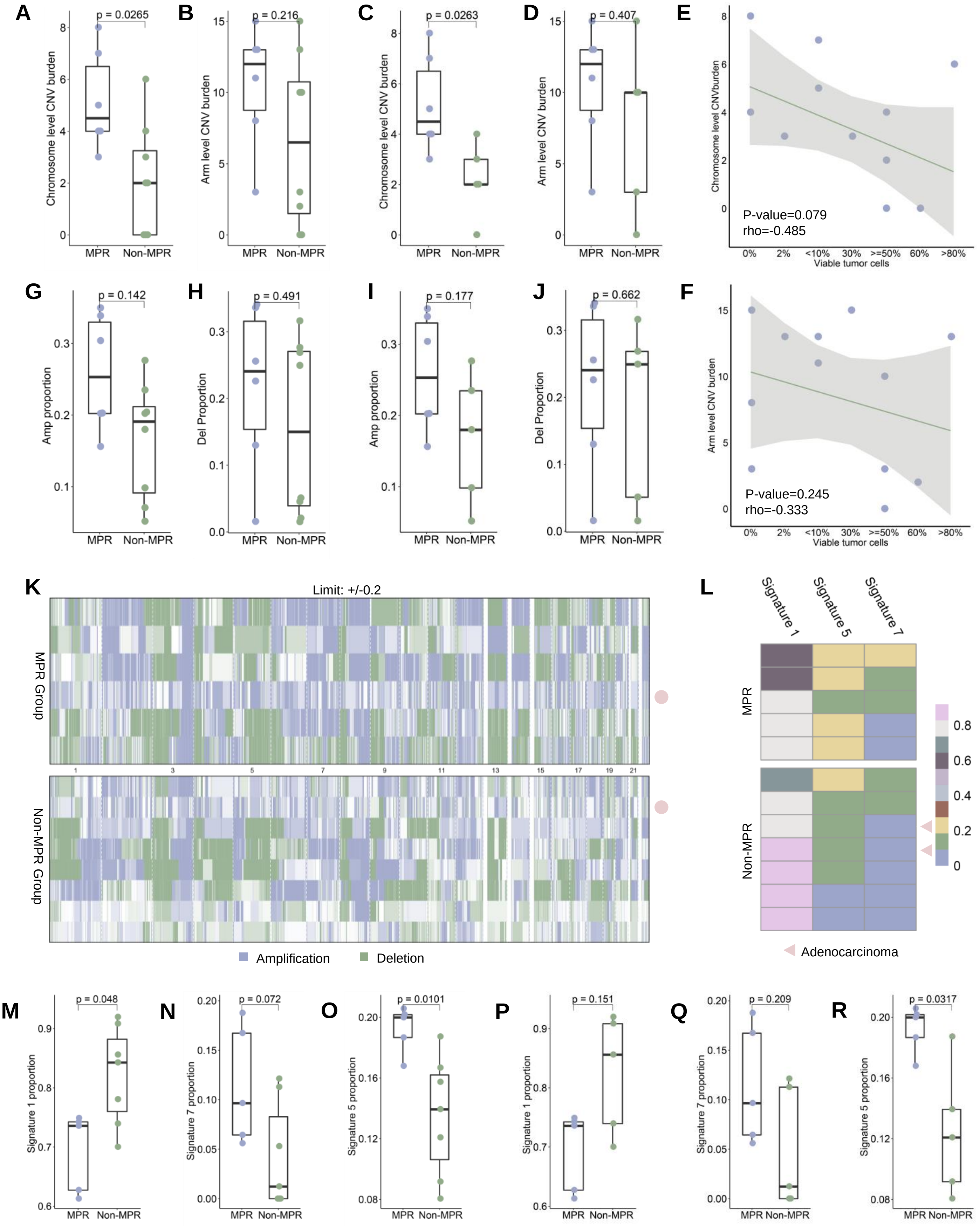

A
p = 0.0265
Chromosome level CNV burden
MPR
Non-MPR
B
p = 0.216
Arm level CNV burden
C
p = 0.0263
D
p = 0.407
E
Chromosome level CNVburden
P-value=0.079
rho=-0.485
Viable tumor cells
0%
2%
<10%
30%
>=50%
60%
>80%
G
p = 0.142
Amp proportion
H
p = 0.491
Del Proportion
I
p = 0.177
J
p = 0.662
F
Arm level CNV burden
P-value=0.245
rho=-0.333
K
Limit: +/-0.2
MPR Group
Non-MPR Group
Amplification
Deletion
L
Signature 1
Signature 5
Signature 7
MPR
Non-MPR
Adenocarcinoma
M
p = 0.048
Signature 1 proportion
N
p = 0.072
Signature 7 proportion
O
p = 0.0101
Signature 5 proportion
P
p = 0.151
Q
p = 0.209
R
p = 0.0317

Suppl. Fig. 3

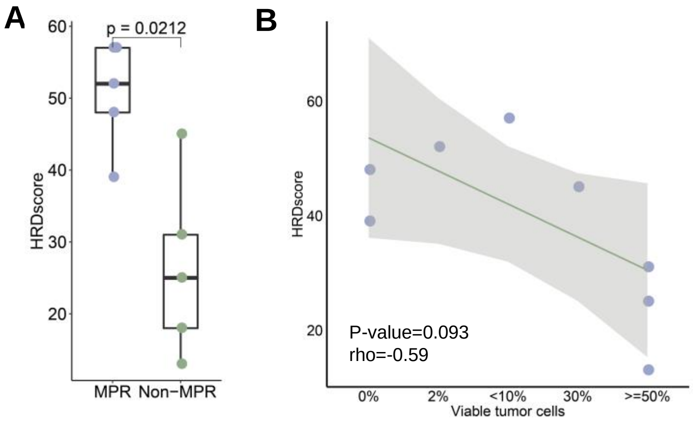
A
p = 0.0212
60
50
40
30
20
HRDscore
MPR
Non−MPR
B
60
40
20
HRDscore
P-value=0.093
rho=-0.59
0%
2%
<10%
30%
>=50%
Viable tumor cells

Suppl. Fig. 4

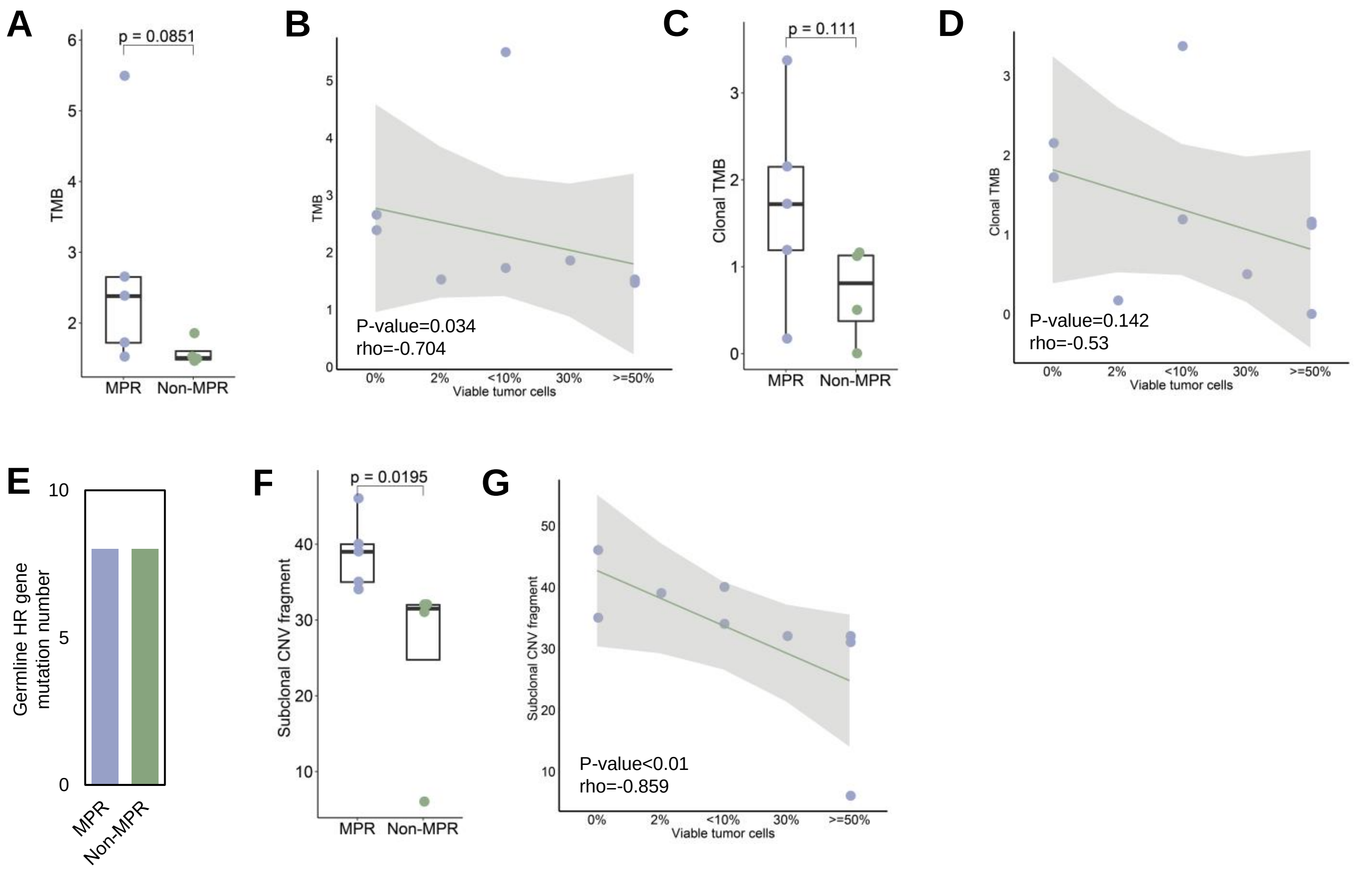
A
p = 0.0851
TMB
MPR
Non-MPR
B
TMB
P-value=0.034
rho=-0.704
0%
2%
<10%
30%
>=50%
Viable tumor cells
C
p = 0.111
Clonal TMB
MPR
Non-MPR
D
Clonal TMB
P-value=0.142
rho=-0.53
Viable tumor cells
E
Germline HR gene mutation number
MPR
Non-MPR
F
p = 0.0195
Subclonal CNV fragment
MPR
Non-MPR
G
Subclonal CNV fragment
P-value<0.01
rho=-0.859
Viable tumor cells

Suppl. Fig. 5

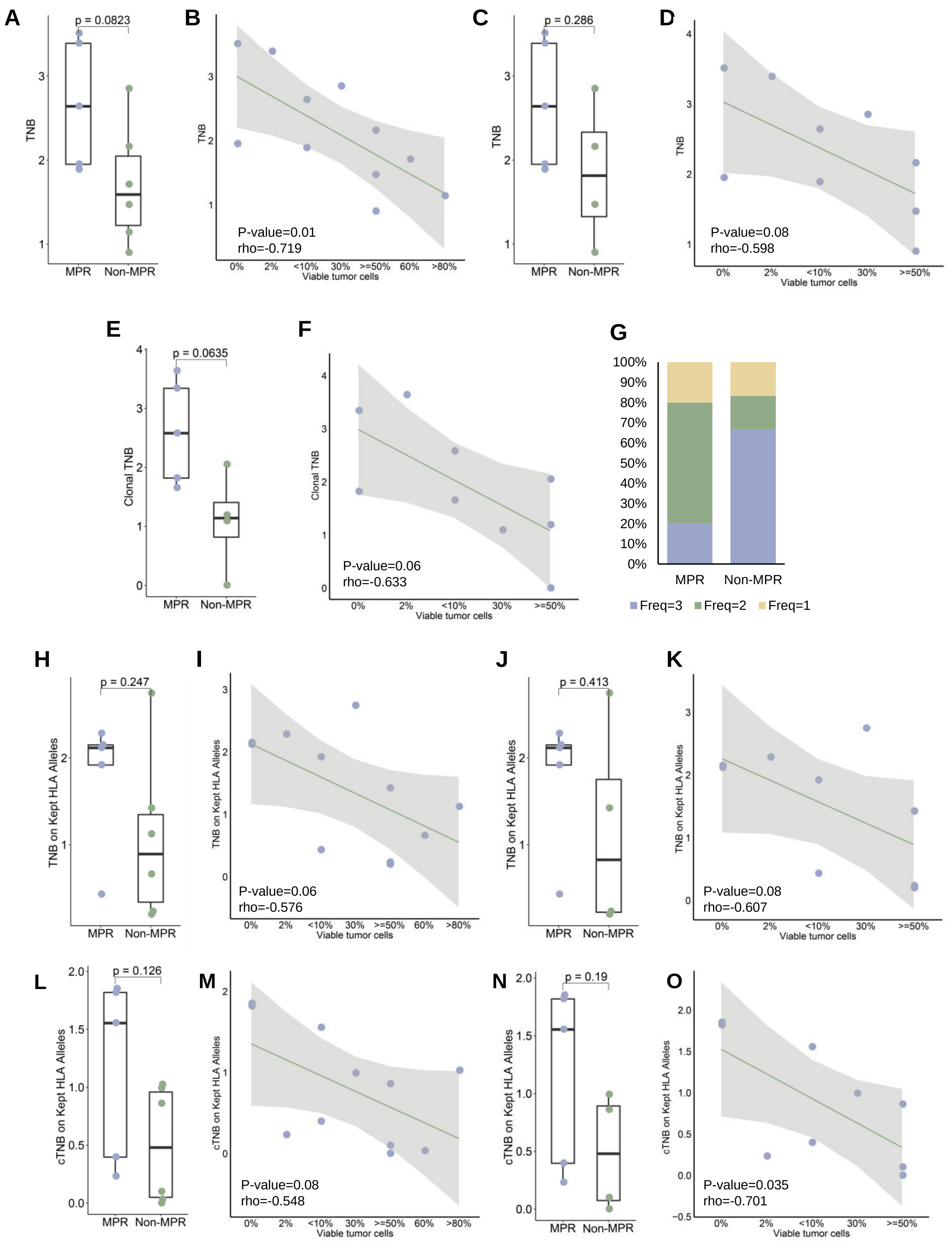
A
p = 0.0823
TNB
MPR
Non-MPR
B
TNB
P-value=0.01
rho=-0.719
0%
2%
<10%
30%
>=50%
60%
>80%
Viable tumor cells
C
p = 0.286
TNB
MPR
Non-MPR
D
TNB
P-value=0.08
rho=-0.598
0%
2%
<10%
30%
>=50%
Viable tumor cells
E
p = 0.0635
Clonal TNB
MPR
Non-MPR
F
Clonal TNB
P-value=0.06
rho=-0.633
0%
2%
<10%
30%
>=50%
Viable tumor cells
G
100%
90%
80%
70%
60%
50%
40%
30%
20%
10%
0%
MPR
Non-MPR
Freq=3
Freq=2
Freq=1
H
p = 0.247
TNB on Kept HLA Alleles
MPR
Non-MPR
I
TNB on Kept HLA Alleles
P-value=0.06
rho=-0.576
0%
2%
<10%
30%
>=50%
60%
>80%
Viable tumor cells
J
p = 0.413
TNB on Kept HLA Alleles
MPR
Non-MPR
K
TNB on Kept HLA Alleles
P-value=0.08
rho=-0.607
0%
2%
<10%
30%
>=50%
Viable tumor cells
L
p = 0.126
cTNB on Kept HLA Alleles
MPR
Non-MPR
M
cTNB on Kept HLA Alleles
P-value=0.08
rho=-0.548
0%
2%
<10%
30%
>=50%
60%
>80%
Viable tumor cells
N
p = 0.19
cTNB on Kept HLA Alleles
MPR
Non-MPR
O
cTNB on Kept HLA Alleles
P-value=0.035
rho=-0.701
0%
2%
<10%
30%
>=50%
Viable tumor cells

Suppl. Fig. 6

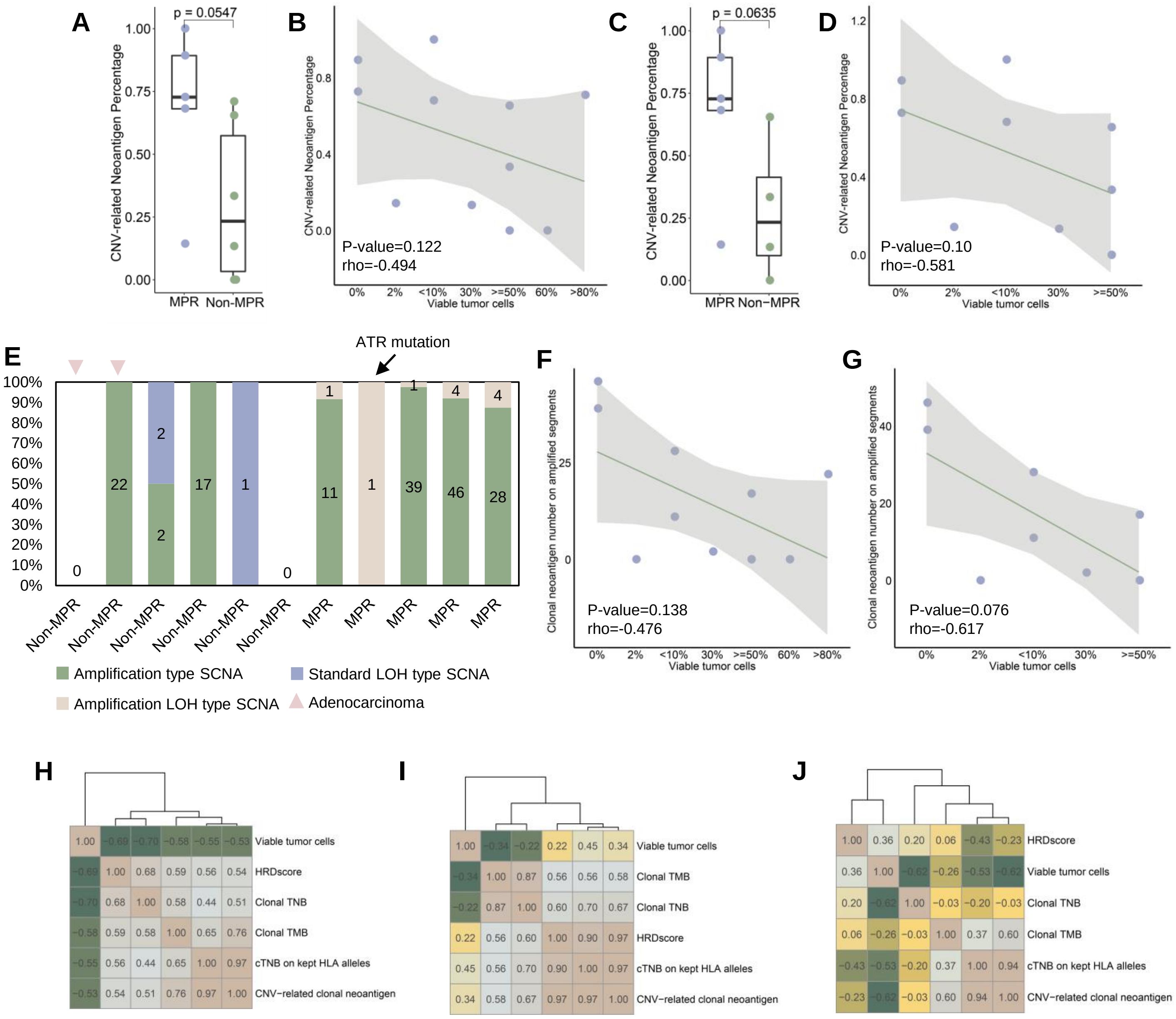
A
p = 0.0547
CNV-related Neoantigen Percentage
MPR
Non-MPR
B
CNV-related Neoantigen Percentage
P-value=0.122
rho=-0.494
Viable tumor cells
C
p = 0.0635
CNV-related Neoantigen Percentage
D
P-value=0.10
rho=-0.581
E
ATR mutation
F
Clonal neoantigen number on amplified segments
P-value=0.138
rho=-0.476
G
P-value=0.076
rho=-0.617
Amplification type SCNA
Standard LOH type SCNA
Amplification LOH type SCNA
Adenocarcinoma
H
I
J
Viable tumor cells
HRDscore
Clonal TNB
Clonal TMB
cTNB on kept HLA alleles
CNV−related clonal neoantigen

Suppl. Fig. 7

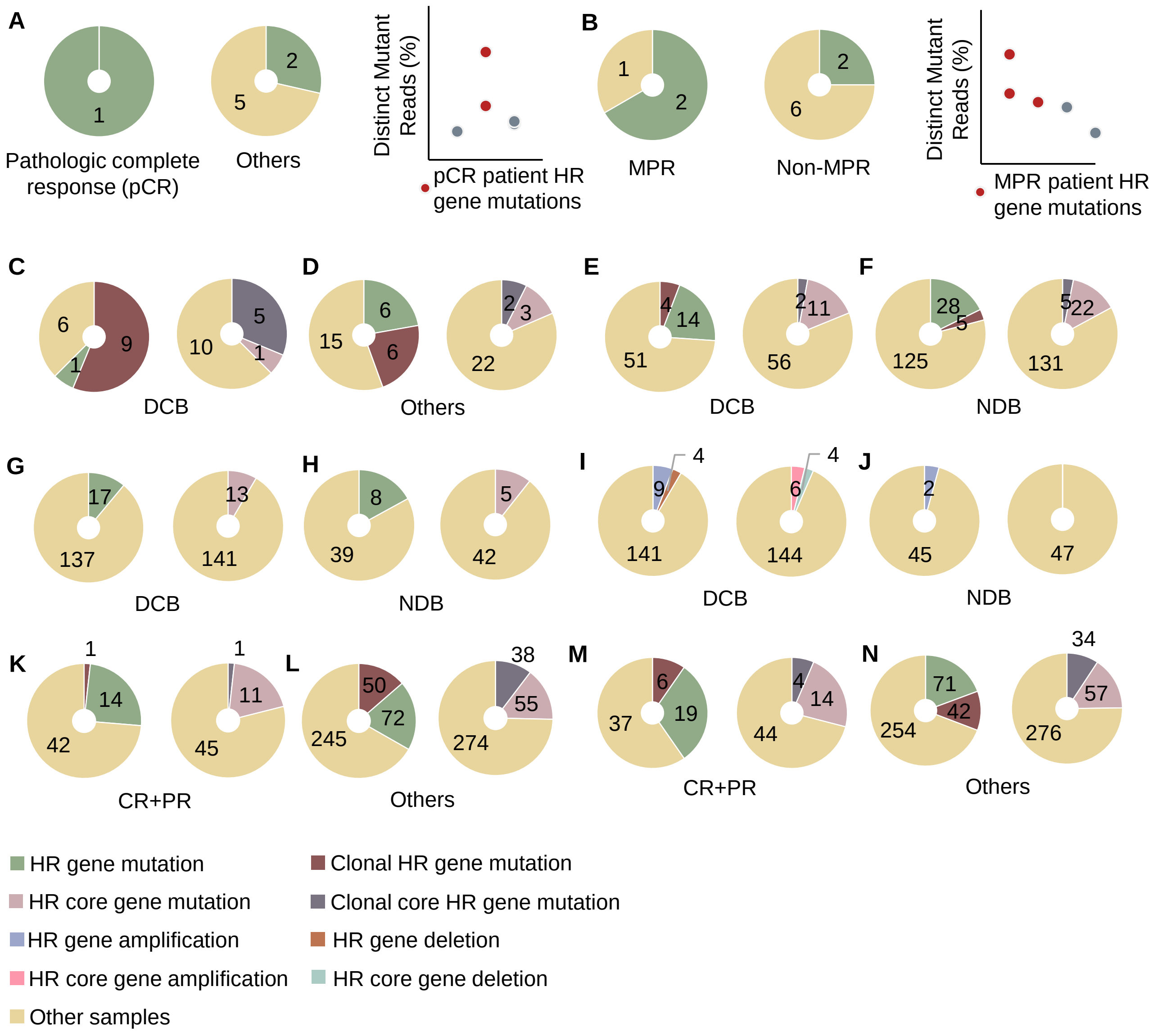
A
1
Pathologic complete response (pCR)
2
5
Others
Distinct Mutant Reads (%)
pCR patient HR gene mutations
B
1
2
MPR
2
6
Non-MPR
Distinct Mutant Reads (%)
MPR patient HR gene mutations
C
6
9
1
10
5
1
DCB
D
15
6
6
2
3
22
Others
E
4
14
51
2
11
56
DCB
F
28
5
125
5
22
131
NDB
G
17
137
13
141
DCB
H
8
39
5
42
NDB
I
4
9
141
4
6
144
DCB
J
2
45
47
NDB
K
1
14
42
1
11
45
CR+PR
L
50
72
245
38
55
274
Others
M
6
19
37
4
14
44
CR+PR
N
71
42
254
34
57
276
Others
HR gene mutation
Clonal HR gene mutation
HR core gene mutation
Clonal core HR gene mutation
HR gene amplification
HR gene deletion
HR core gene amplification
HR core gene deletion
Other samples

Suppl. Fig. 8

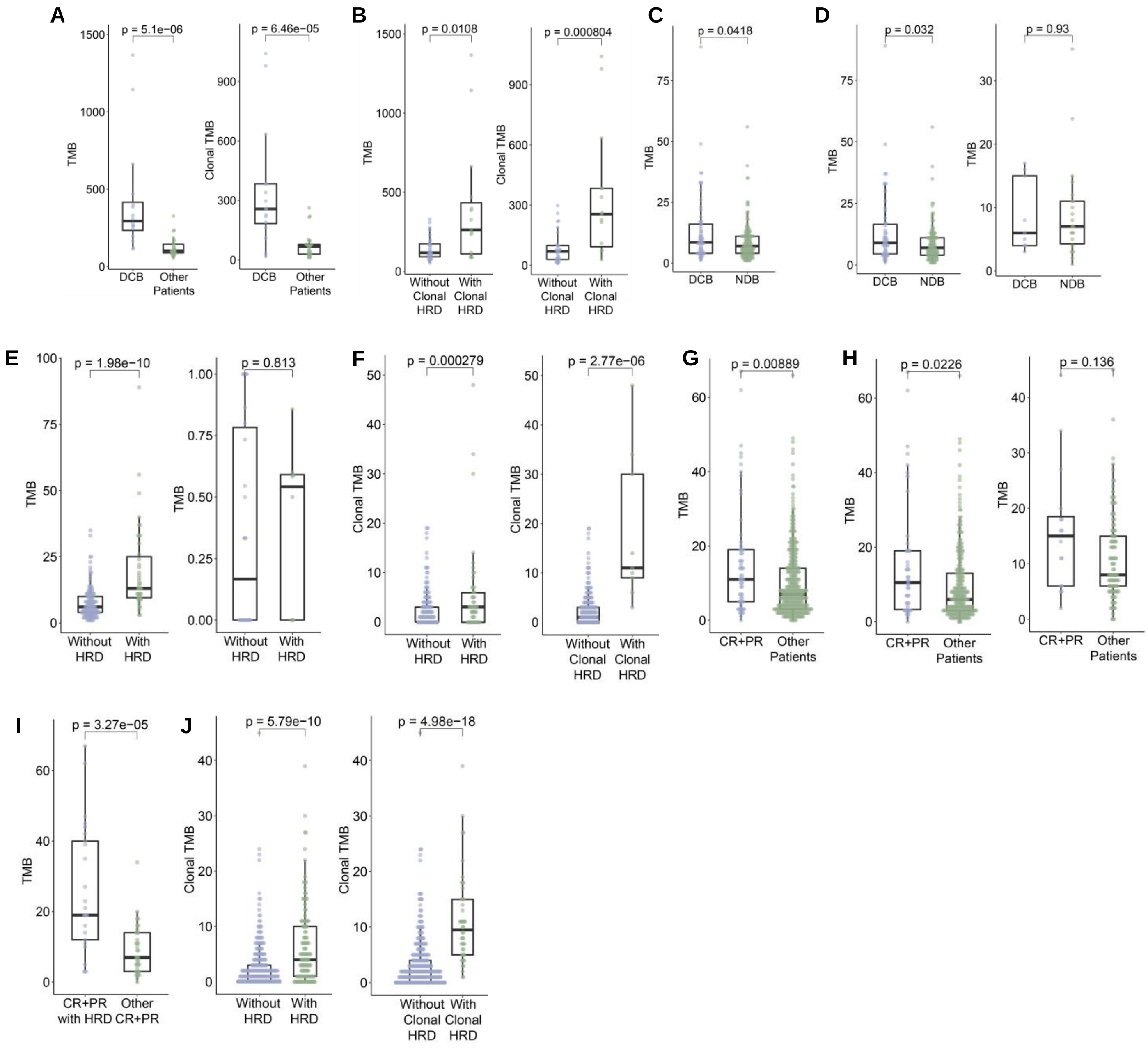
A
p = 5.1e−06
TMB
DCB
Other Patients
p = 6.46e−05
Clonal TMB
DCB
Other Patients
B
p = 0.0108
TMB
Without Clonal HRD
With Clonal HRD
p = 0.000804
Clonal TMB
Without Clonal HRD
With Clonal HRD
C
p = 0.0418
TMB
DCB
NDB
D
p = 0.032
TMB
DCB
NDB
p = 0.93
TMB
DCB
NDB
E
p = 1.98e−10
TMB
Without HRD
With HRD
p = 0.813
TMB
Without HRD
With HRD
F
p = 0.000279
Clonal TMB
Without HRD
With HRD
p = 2.77e−06
Clonal TMB
Without Clonal HRD
With Clonal HRD
G
p = 0.00889
TMB
CR+PR
Other Patients
H
p = 0.0226
TMB
CR+PR
Other Patients
p = 0.136
TMB
CR+PR
Other Patients
I
p = 3.27e−05
TMB
CR+PR with HRD
Other CR+PR
J
p = 5.79e−10
Clonal TMB
Without HRD
With HRD
p = 4.98e−18
Clonal TMB
Without Clonal HRD
With Clonal HRD

Suppl. Fig. 9

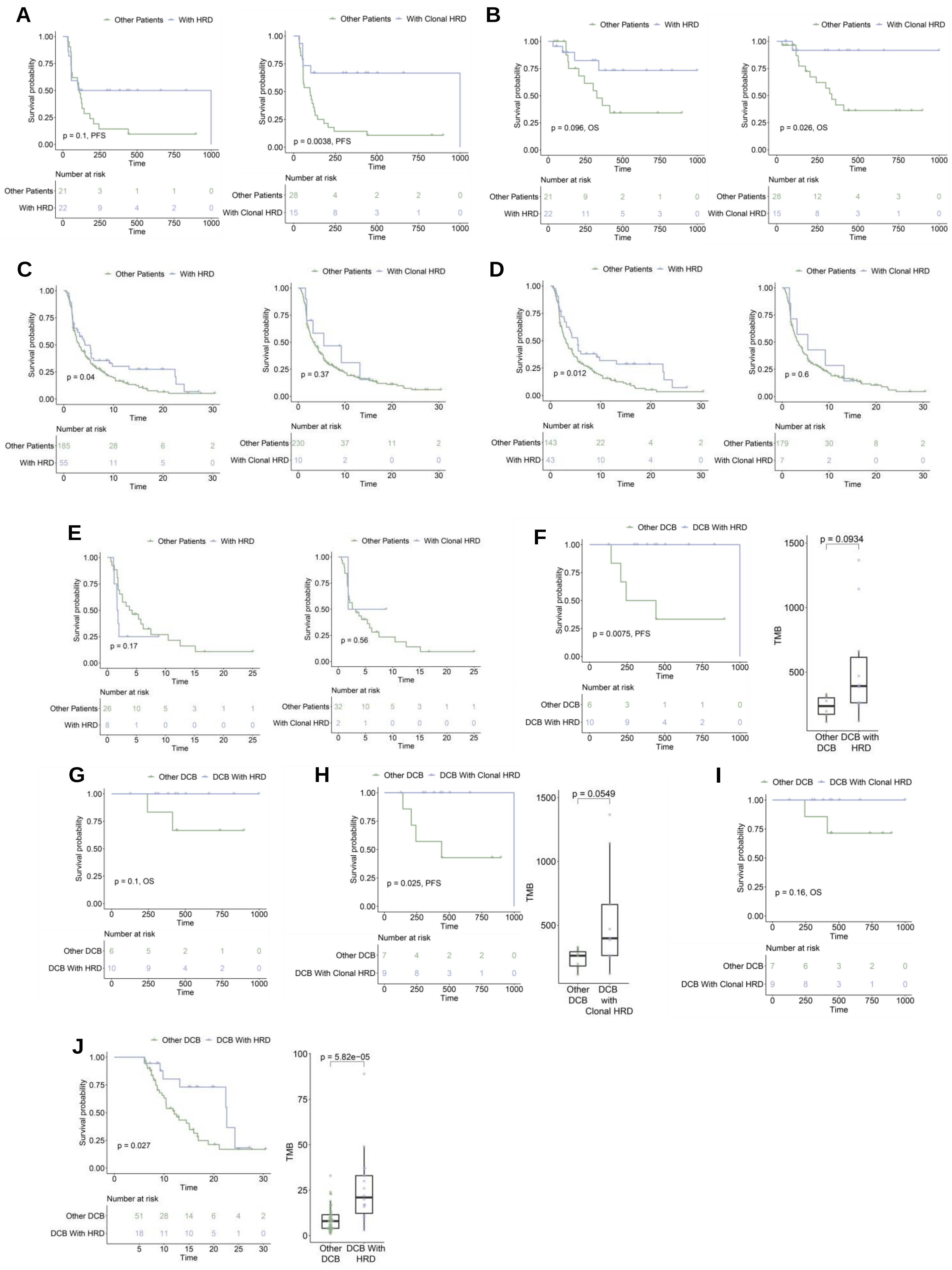
A
Other Patients With HRD
Survival probability
p = 0.1, PFS
Time
Number at risk
Other Patients 21 3 1 1 0
With HRD 22 9 4 2 0
Other Patients With Clonal HRD
p = 0.0038, PFS
Other Patients 28 4 2 2 0
With Clonal HRD 15 8 3 1 0
B
Other Patients With HRD
p = 0.096, OS
Other Patients 21 9 2 1 0
With HRD 22 11 5 3 0
Other Patients With Clonal HRD
p = 0.026, OS
Other Patients 28 12 4 3 0
With Clonal HRD 15 8 3 1 0
C
Other Patients With HRD
p = 0.04
Other Patients 185 28 6 2
With HRD 55 11 5 0
Other Patients With Clonal HRD
p = 0.37
Other Patients 230 37 11 2
With Clonal HRD 10 2 0 0
D
Other Patients With HRD
p = 0.012
Other Patients 143 22 4 2
With HRD 43 10 4 0
Other Patients With Clonal HRD
p = 0.6
Other Patients 179 30 8 2
With Clonal HRD 7 2 0 0
E
Other Patients With HRD
p = 0.17
Other Patients 26 10 5 3 1 1
With HRD 8 1 0 0 0 0
Other Patients With Clonal HRD
p = 0.56
Other Patients 32 10 5 3 1 1
With Clonal HRD 2 1 0 0 0 0
F
Other DCB DCB With HRD
p = 0.0075, PFS
Other DCB 6 3 1 1 0
DCB With HRD 10 9 4 2 0
p = 0.0934
TMB
Other DCB
DCB with HRD
G
Other DCB DCB With HRD
p = 0.1, OS
Other DCB 6 5 2 1 0
DCB With HRD 10 9 4 2 0
H
Other DCB DCB With Clonal HRD
p = 0.025, PFS
Other DCB 7 4 2 2 0
DCB With Clonal HRD 9 8 3 1 0
p = 0.0549
TMB
Other DCB
DCB with Clonal HRD
I
Other DCB DCB With Clonal HRD
p = 0.16, OS
Other DCB 7 6 3 2 0
DCB With Clonal HRD 9 8 3 1 0
J
Other DCB DCB With HRD
p = 0.027
Other DCB 51 28 14 6 4 2
DCB With HRD 18 11 10 5 1 0
p = 5.82e−05
TMB
Other DCB
DCB With HRD

Suppl. Fig. 10

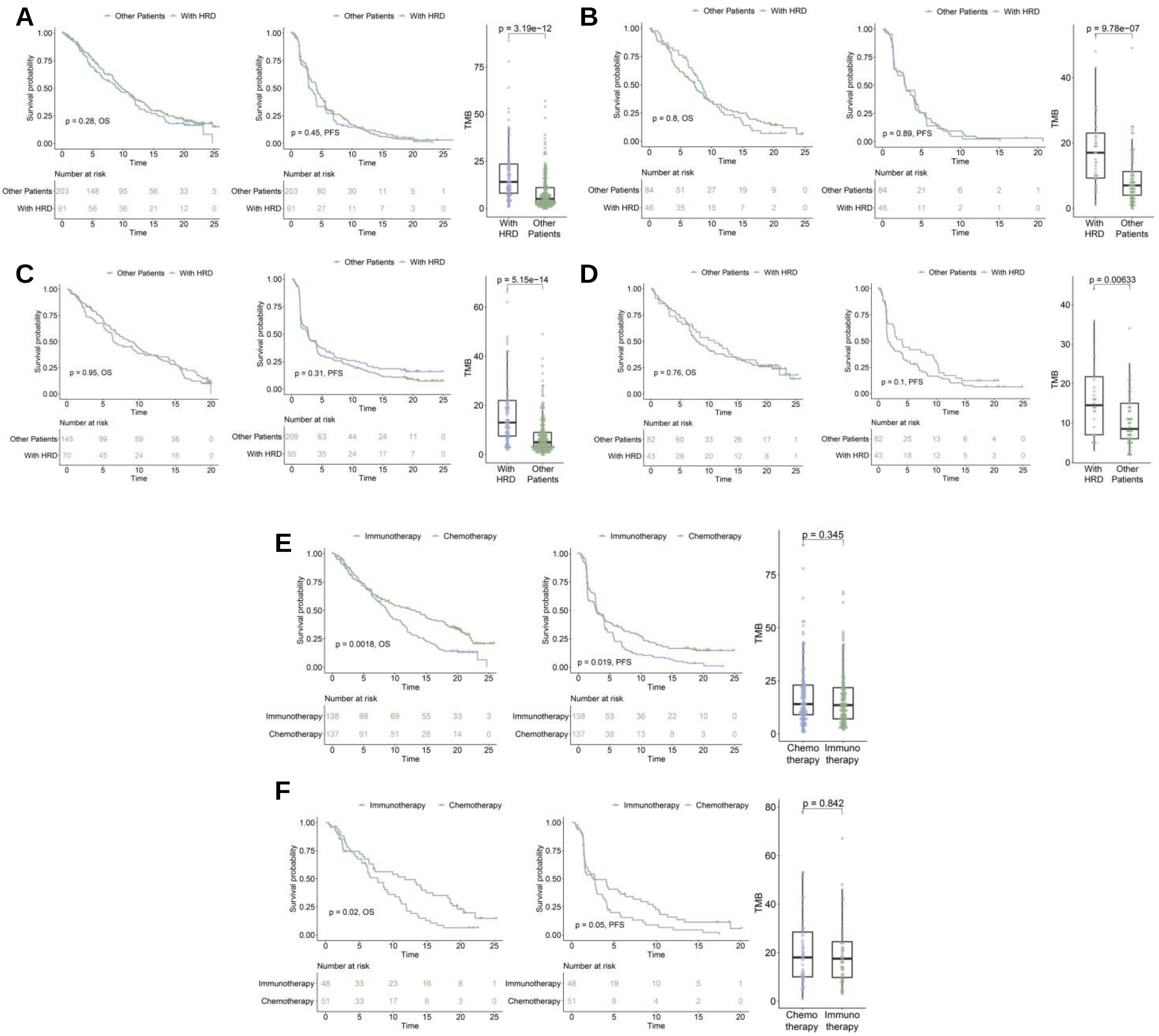
A
Other Patients
With HRD
Survival probability
p = 0.28, OS
p = 0.45, PFS
Time
Number at risk
Other Patients 203 148 95 56 33 5
With HRD 91 56 36 21 12 0
Other Patients 203 80 30 11 5 1
With HRD 91 27 11 7 3 0
p = 3.19e−12
TMB
With HRD
Other Patients
B
p = 0.8, OS
p = 0.89, PFS
Other Patients 84 51 27 19 9 0
With HRD 46 35 15 7 2 0
Other Patients 84 21 6 2 1
With HRD 46 11 2 1 0
p = 9.78e−07
C
p = 0.95, OS
p = 0.31, PFS
Other Patients 145 99 59 38 0
With HRD 70 45 24 18 0
Other Patients 209 63 44 24 11 0
With HRD 95 35 24 17 7 0
p = 5.15e−14
D
p = 0.76, OS
p = 0.1, PFS
Other Patients 82 60 33 26 17 1
With HRD 43 28 20 12 8 1
Other Patients 82 25 13 6 4 0
With HRD 43 18 12 5 3 0
p = 0.00633
E
Immunotherapy
Chemotherapy
p = 0.0018, OS
p = 0.019, PFS
Immunotherapy 138 98 69 55 33 3
Chemotherapy 137 91 51 28 14 0
Immunotherapy 138 53 36 22 10 0
Chemotherapy 137 38 13 8 3 0
p = 0.345
Chemo therapy
Immuno therapy
F
p = 0.02, OS
p = 0.05, PFS
Immunotherapy 48 33 23 16 8 1
Chemotherapy 51 33 17 6 3 0
Immunotherapy 48 19 10 5 1
Chemotherapy 51 9 4 2 0
p = 0.842

Suppl. Fig. 11

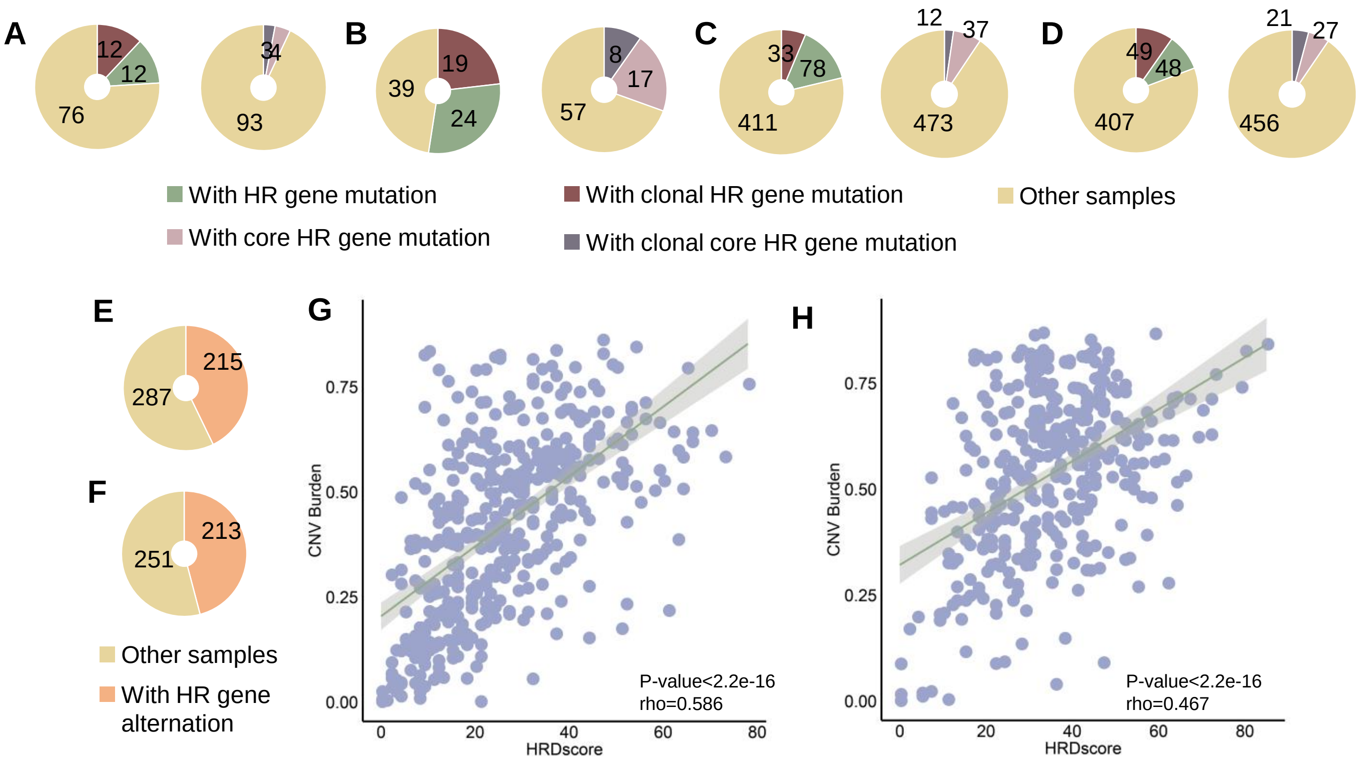
A
12
12
76
3
4
93
B
19
39
24
8
17
57
C
33
78
411
12
37
473
D
49
48
407
21
27
456
With HR gene mutation
With core HR gene mutation
With clonal HR gene mutation
With clonal core HR gene mutation
Other samples
E
215
287
F
213
251
Other samples
With HR gene alternation
G
CNV Burden
0.00
0.25
0.50
0.75
HRDscore
0
20
40
60
80
P-value<2.2e-16
rho=0.586
H
CNV Burden
0.00
0.25
0.50
0.75
HRDscore
0
20
40
60
80
P-value<2.2e-16
rho=0.467
