# Supplementary material for: Homologous recombination deficiency (HRD) can predict the therapeutic outcomes of immuno-neoadjuvant therapy in NSCLC patients
Source: J Hematol Oncol. 2022 May 18;15:62. doi: 10.1186/s13045-022-01283-7 (PMC9118717; doi:10.1186/s13045-022-01283-7)
Supplement: Supplementary file 4 — Additional file 4: Supplementary Materials and methods. [file 13045_2022_1283_MOESM4_ESM.docx]

**Supplementary Materials and methods**

**Patient enrollment, specimen collection and sequencing data acquisition**

A total of 3 adenocarcinoma and 11 squamous cell carcinoma NSCLC patients were accrued from 2018 to 2020 at Shanghai Chest Hospital with immuno-neoadjuvant therapy treatment and surgical resection. The demographics of recruited patients and treatment characteristics were listed in Additional file 1: Table S1. Pre-therapy FFPE/fresh tissues were biopsied and peripheral blood mononuclear cell (PBMC) were isolated from blood samples for genomic DNA extraction by AllPrep DNA/RNA FFPE/Mini Kit (Qiagen) or CWE9600 Blood DNA kit (CWBiotech, China) and subsequent 100bp paired-end WES library construction. Final sequencing data was acquired by Geneplus-2000 sequencing platform (Geneplus, Beijing, China). Metrics including total read volume, Q30 percentage, GC content and duplication rate were used for quality assessment. Besides, instead of OS requiring a much longer follow-up, we utilized MPR, which is significantly predictive of long-term OS in neoadjuvant therapy^1^, as a surrogate endpoint for therapy response evaluation. Patients enrolled were categorized as MPR when the viable tumor cells in the resection specimen was fewer than 10% after treatment, in line with the immune-related pathologic response criteria (irPRC) proposed by Cottrell et al.^2^

**Mutation calling, copy number alternation identification and signature analysis**

After BWA (version 0.7.10) read alignment on hg19 reference genome and sample coverage filtration, MuTect (version 1.1.4) in GATK (version 4.0) pipeline identified somatic single nucleotide variants (SNVs), small insertions and deletions (InDels) by comparing tissue/PBMC specimens while GATK prioritized segment-level somatic copy number alterations (SCNA). Two rounds of filtration were conducted on SNVs and InDels, including reliability filtering by retaining variants with low frequency (<=0.01) in population, non-zero variant allele frequency (>=0.01), more supporting reads (>=3) and functional alternations (nonsense, in-frame/frame-shift small insertions and deletions, mutations in canonical splice-sites). Cancer associated genes^3^ were further extracted to conduct cancer-related filtration. As for the germline mutations, the SNPs and InDels were identified following workflows in GATK. Annotations including mutation types, resided transcripts and allele frequencies of the healthy population were conducted by Ensembl Varient Effector Predictor^4^. Functional mutations predicted as damaging and deleterious by PolyPhen^5^ and SIFT^6^ tools were initially retained. Later mutations with allele frequency>0.01 in all as well as the East Asian population from ExAC, 1000G and gnomAD databases were discarded in the further analyses. As for mutational signatures, unfiltered somatic SNVs were fed into MutationalPatterns^7^ tool to derive their relative contribution to the base substitution spectrum and absolute contribution of COSMIC v3 SBS (single base substitution) mutational signatures. COSMIC v3 DBS (double base substitution) and ID (InDel) signature absolute exposures were quantified by Sigminer^8^. Quantification of existing seven SCNA signatures in curated samples was obtained by CNsignatures^9^.

**Intratumor heterogeneity measurement and SNV/SCNA clonality annotation**

The genomic diversification denoted by intratumor heterogeneity (ITH) were measured at both somatic SNV and SCNA scope. The mutant-allele tumor heterogeneity (MATH) score^10^ was calculated using sample-wise variant allele frequency (VAF) on filtered mutations. SNV and SCNA data were jointly considered to estimate the cancer cell fraction (CCF), cancer ploidy, tumor purity and rescaled copy ratio by ABSOLUTE^11^ followed by model selection manual review. SNV was further annotated as clonal if its CCF upper 95% confidence interval>=1 and the clonal mutation probability was higher. Between-allelic rescaled copy number ratio comparison was conducted to exclude copy neutral LOH (CNLOH) segments and annotate SCNA segments using allelic subclonal information from ABSOLUTE outputs.

**HRD event quantification and HR pathway gene analysis**

A NGS-based R package scarHRD^12^ was used to calculate three HRD metrics including telomeric allelic imbalance (TAI), loss off heterozygosity (LOH) and number of large-scale transitions (LST). Genes from HR pathway and core pathway^13^ were collected for the intersection with clonality-annotated SNV/germline mutations/SCNA.

**Neoantigen identification and HLA-LOH event evaluation**

pVACseq^14^ was exploited for neoantigen prediction and the TNB was normalized as per-megabase neoantigen that passed filtration. The HLA-LOH event occurrence was determined by LOHHLA tool^15^ and the alleles with allelic imbalance p-value<0.05 was retained.

**Public data curation for validation**

For multi-cohort data validation on the discriminative power of HRD in clinical treatments, data were retrieved from multiple immunotherapy and chemotherapy lung cancer datasets. More specifically, based on treatment scheme, the curated datasets were classified into neoadjuvant immunotherapy, immunotherapy and multi-therapy categories. As for the neoadjuvant immunotherapy group, results from two studies utilizing WES in genomic characteristic profiling on NSCLC patients were collected^16,17^ (denoted as N Engl J Med. 2018 and J Immunother Cancer. 2020). As for immunotherapy group, one WES dataset^18^ denoted as Nat Genet. 2018 and one targeted sequencing dataset^19^ namely J Clin Oncol. 2018 were included. As a category in which datasets contain patients received distinct therapy types, targeted sequencing results on tissue^20^ (named as Cancer Discov. 2017) and blood^21^ samples (named as Nat. Med. 2018) were collected for treatment level comparisons in multiple therapy categories. Based on the sequencing result availability, alternation frequencies of HR pathway genes (i.e. HRD event) were calculated using functional mutations, mutation burdens were compared and survival differences were measured. Additionally, multiracial datasets^22,23^ named as Sci. Rep. 2015, J Thorac Oncol. 2020, TCGA-LUAD (The Cancer Genome Atlas, lung adenocarcinoma) and TCGA-LUSC (lung squamous cell carcinoma) were collected either from papers or TCGA repository in cBioPortal database^24^ to investigate the alternation frequency of HR pathway genes in treatment-free multiethnic patients. Two datasets^13,25^ containing pan-cancer HRD event analysis on TCGA data were also integrated for HRD frequency statistics.

**Statistical analysis**

Both the two-sided and one-side Wilcoxon rank-sum tests were used for evaluating the group-level difference of continuous values including mutation relative contribution, SCNA, HRD levels, TMB and TNB between MPR and Non-MPR patients. When the group-wise comparisons were conducted on categorical data, Fisher’s exact test was applied. Correlations between genetic metrics and percentage of viable tumor cells were measured by Spearman's correlation coefficient. As for survival analysis, the Kaplan-Meier (K-M) survival curves were generated by survminer package^26^ which applied log-rank test for survival time comparisons.

**References**

1. Weissferdt, A. *et al.* Agreement on Major Pathological Response in NSCLC Patients Receiving Neoadjuvant Chemotherapy. *Clinical Lung Cancer* **21**, 341–348 (2020).

2. Cottrell, T. R. *et al.* Pathologic features of response to neoadjuvant anti-PD-1 in resected non-small-cell lung carcinoma: a proposal for quantitative immune-related pathologic response criteria (irPRC). *Ann Oncol* **29**, 1853–1860 (2018).

3. Angus, L. *et al.* Genomic landscape of metastatic breast cancer and its clinical implications. *Nat Genet* **51**, 1450–1458 (2019).

4. McLaren, W. *et al.* The Ensembl Variant Effect Predictor. *Genome Biology* **17**, 122 (2016).

5. Adzhubei, I., Jordan, D. M. & Sunyaev, S. R. Predicting Functional Effect of Human Missense Mutations Using PolyPhen-2. *Curr Protoc Hum Genet* **0 7**, Unit7.20 (2013).

6. Ng, P. C. & Henikoff, S. SIFT: predicting amino acid changes that affect protein function. *Nucleic Acids Res* **31**, 3812–3814 (2003).

7. Blokzijl, F., Janssen, R., van Boxtel, R. & Cuppen, E. MutationalPatterns: comprehensive genome-wide analysis of mutational processes. *Genome Medicine* **10**, 33 (2018).

8. Wang, S., Tao, Z., Wu, T. & Liu, X.-S. Sigflow: an automated and comprehensive pipeline for cancer genome mutational signature analysis. *Bioinformatics* (2020) doi:10.1093/bioinformatics/btaa895.

9. Macintyre, G. *et al.* Copy-number signatures and mutational processes in ovarian carcinoma. *Nat Genet* **50**, 1262–1270 (2018).

10. Mroz, E. A. & Rocco, J. W. MATH, a novel measure of intratumor genetic heterogeneity, is high in poor-outcome classes of head and neck squamous cell carcinoma. *Oral Oncol* **49**, 211–215 (2013).

11. Carter, S. L. *et al.* Absolute quantification of somatic DNA alterations in human cancer. *Nature Biotechnology* **30**, 413–421 (2012).

12. Sztupinszki, Z. *et al.* Migrating the SNP array-based homologous recombination deficiency measures to next generation sequencing data of breast cancer. *npj Breast Cancer* **4**, 1–4 (2018).

13. Knijnenburg, T. A. *et al.* Genomic and Molecular Landscape of DNA Damage Repair Deficiency across The Cancer Genome Atlas. *Cell Rep* **23**, 239-254.e6 (2018).

14. Hundal, J. *et al.* pVAC-Seq: A genome-guided in silico approach to identifying tumor neoantigens. *Genome Med* **8**, (2016).

15. McGranahan, N. *et al.* Allele-Specific HLA Loss and Immune Escape in Lung Cancer Evolution. *Cell* **171**, 1259-1271.e11 (2017).

16. Forde, P. M. *et al.* Neoadjuvant PD-1 Blockade in Resectable Lung Cancer. *New England Journal of Medicine* **378**, 1976–1986 (2018).

17. Reuss, J. E. *et al.* Neoadjuvant nivolumab plus ipilimumab in resectable non-small cell lung cancer. *J Immunother Cancer* **8**, e001282 (2020).

18. Miao, D. *et al.* Genomic correlates of response to immune checkpoint blockade in microsatellite-stable solid tumors. *Nature Genetics* **50**, 1271–1281 (2018).

19. Rizvi, H. *et al.* Molecular Determinants of Response to Anti-Programmed Cell Death (PD)-1 and Anti-Programmed Death-Ligand 1 (PD-L1) Blockade in Patients With Non-Small-Cell Lung Cancer Profiled With Targeted Next-Generation Sequencing. *J Clin Oncol* **36**, 633–641 (2018).

20. Jordan, E. J. *et al.* Prospective Comprehensive Molecular Characterization of Lung Adenocarcinomas for Efficient Patient Matching to Approved and Emerging Therapies. *Cancer Discov* **7**, 596–609 (2017).

21. Gandara, D. R. *et al.* Blood-based tumor mutational burden as a predictor of clinical benefit in non-small-cell lung cancer patients treated with atezolizumab. *Nat Med* **24**, 1441–1448 (2018).

22. Li, C. *et al.* Whole Exome Sequencing Identifies Frequent Somatic Mutations in Cell-Cell Adhesion Genes in Chinese Patients with Lung Squamous Cell Carcinoma. *Sci Rep* **5**, 14237 (2015).

23. Arauz, R. F. *et al.* Whole-Exome Profiling of NSCLC Among African Americans. *J Thorac Oncol* **15**, 1880–1892 (2020).

24. Gao, J. *et al.* Integrative analysis of complex cancer genomics and clinical profiles using the cBioPortal. *Science signaling* **6**, pl1–pl1 (2013).

25. Sinha, S. *et al.* Higher prevalence of homologous recombination deficiency in tumors from African Americans versus European Americans. *Nat Cancer* **1**, 112–121 (2020).

26. Kassambara, A., Kosinski, M., Biecek, P. & Fabian, S. Package ‘survminer’. *Drawing Survival Curves using ‘ggplot2’(R package version 03 1)* (2017).
